# PD-1/LAG-3 bispecific antibody potentiates T cell activation and increases antitumor efficacy

**DOI:** 10.3389/fimmu.2022.1047610

**Published:** 2022-11-28

**Authors:** Ning Shi, Yangyihua Zhou, Yujun Liu, Ran Zhang, Xingjun Jiang, Caiping Ren, Xiang Gao, Longlong Luo

**Affiliations:** ^1^ National Health Commission (NHC) Key Laboratory of Carcinogenesis, Department of Neurosurgery, Xiangya Hospital, Central South University, Changsha, Hunan, China; ^2^ State Key Laboratory of Toxicology and Medical Countermeasures, Institute of Pharmacology and Toxicology, Beijing, China; ^3^ National Clinical Research Center for Geriatric Disorders, Xiangya Hospital, Central South University, Changsha, Hunan, China; ^4^ Department of Neurosurgery, Xiangya Hospital, Central South University, Changsha, Hunan, China; ^5^ Cancer Research Institute, School of Basic Medicine, Central South University, Changsha, Hunan, China; ^6^ Hunan Normal University School of Medicine, Changsha, Hunan, China

**Keywords:** immune checkpoint, PD-1, LAG-3, bispecific antibody, cancer immunotherapy

## Abstract

Several clinical studies demonstrate that there exist other immune checkpoints overexpressed in some PD-1 inhibitor-resistant tumor patients. Among them, Lymphocyte-activation gene 3 (LAG-3) is one of the important immune checkpoint molecules and has been clinically demonstrated to have synergistic anti-tumor effects in combination with PD-1 antibody. In this study, we designed a novel ‘knob-in-hole’ PD-1/LAG-3 bispecific antibody (BsAb) YG-003D3. In conclusion, the BsAb maintained the similar affinity and thermal stability to the parental antibody, and the BsAb structure can be independent of each other in the process of double-target recognition, and the recognition activity will not be affected. Moreover, the BsAb can not only target PD-1 and LAG-3 on single cell simultaneously, but also bridge the two kinds of cells expressing PD-1 and LAG-3, so as to release the ‘brake system of immune checkpoints’ and activate immune cells to exert anti-tumor effects more effectively. Especially in the PBMCs activation assay, YG-003D3 induced stronger IFN-γ, IL-6, and TNF-α secretion compared to anti-PD-1 or anti-LAG-3 single drug group or even combined drug group. In the tumor killing experiment of PBMC *in vitro*, YG-003D3 has a better ability to activate PBMC to kill tumor cells than anti-PD-1 or anti-LAG-3 single drug group or even combined drug group, and the killing rate is as high as 20%. In a humanized PD-1/LAG-3 transgenic mouse subcutaneous tumor-bearing model, YG-003D3 showed good anti-tumor activity, even better than that of the combination group at the same molar concentration. Further studies have shown that YG-003D3 could significantly alter the proportion of immune cells in the tumor microenvironment. In particular, the proportion of CD45^+^, CD3^+^ T, CD8^+^ T cells in tumor tissue and the proportion of CD3^+^ T, CD8^+^ T, CD4^+^ T cells in peripheral blood were significantly increased. These results suggest that YG-003D3 exerts a potent antitumor effect by activating the body ‘s immune system. In summary, the BsAb YG-003D3 has good anti-tumor activity, which is expected to become a novel drug candidate for cancer immunotherapy.

## Introduction

Cancer immunotherapy is a new generation of anti-tumor therapy. Its basic idea is to activate the human immune system by relieving the immunosuppression of the tumor microenvironment, improving the antigen presentation function of dendritic cells, and promoting the production of protective T cells, to identify and kill the cancer cells (1–3). PD-1 is one of the hottest immune checkpoint molecules in recent years. The interaction between PD-1 and its ligand PD-L1 inhibits T cell activity and helps tumor cells escape the surveillance of the human immune system (4–6). Many studies have shown that blocking the PD-1/PD-L1 pathway can effectively enhance the anti-tumor immune effect (7–9). At present, a variety of targeted PD-1/PD-L1 antibody drugs have been approved for the market, such as Nivolumab, Atezolizumab, etc. However, Checkpoint inhibitors targeting the PD-1/PD-L1 pathway have shown limited (clinically validated effective response rate of approximately 20 percent) (10–13). One important reason is that resistance to cancer immunotherapy can be mediated by additional immune checkpoints (14). There are intense ongoing efforts to explore new immune checkpoint molecules and a related drug and improve the response rate of PD-1 antibody therapy in a drug-resistant population.

Among the potential immune checkpoint molecules, LAG-3 has become a possible candidate target based on positive results in preclinical studies and clinical trials (15–18). LAG-3 is an inhibitory regulator, which can regulate the signaling pathways of T lymphocytes and antigen-presenting cells (APCs) and plays an important role in adaptive immune response (19–22). Studies have shown that inhibition of LAG-3 can restore the exhausted T cells to regain cytotoxic activity and reduce regulating T cells’ immune suppression function, thereby enhancing the killing effect on tumors (23). Moreover, some studies have shown that PD-1 and LAG-3 are co-expressed in the tumor immune microenvironment, which jointly mediates the immune escape effect of tumor cells (20, 23–26). Especially, BMS has published a phase II/III study (RELATIVITY-047) in 2021. The results showed that the fixed dose of Relatlimab (Anti-LAG-3 monoclonal antibody) combined with Nivolumab (Anti-PD-1 monoclonal antibody) in the treatment of metastatic or unresectable melanoma can significantly improve the progression-free survival (PFS) of patients compared with Nivolumab monotherapy (10.1 months vs 4.6 months) (15, 16). The above evidence shows that the LAG-3 is somehow involved in PD-1 antibody drug resistance (15, 16). Therefore, blocking PD-1 and LAG-3 signal pathways simultaneously may bring a better anti-tumor effect. Combined administration or bispecific antibodies (BsAbs) drugs can achieve dual target blockade, but BsAbs have more advantages in drug space-time distribution and cell cross-linking. Therefore, several BsAbs targeting PD-(L)1 and LAG-3 are in the pre-clinical or early clinical research stage, such as IBI323, Tebotelimab, etc. (27–29). In preclinical studies, IBI323(a PD-L1/LAG-3 bispecific antibody) (27), Tebotelimab(a PD-1/LAG-3 DART bispecific antibody) (28, 29), have shown better *in vitro* and *in vivo* efficacy than anti-LAG-3 monoclonal antibody and anti-PD-L1/anti-PD-1 monoclonal antibody. We have previously reported an anti-PD-1 antibody, Fv78 (30), and also screened a human anti-LAG-3 antibody, MIL80, which has similar biological activity to the market antibody nivolumab or clinical antibody relatlimab. to expand the application of the above-mentioned antibodies, we designed a ‘knob-in-hole’ BsAbs YG-003D3 based on Fv78 and MIL-80. We further designed *in vitro* and *in vivo* experiments to evaluate the potential anti-tumor activity of bispecific antibodies and further studied the advantages and pharmacological characteristics of bispecific antibodies compared with monotherapy or combination therapy of both drugs. We show that YG-003D3 can maintain the binding activity with LAG-3 and PD-1, especially in the process of antigen recognition, it can bind LAG-3 and PD-1 at the same time without steric hindrance effect. Moreover, we found that the binding ability of YG-003D3 to PBMCs (theoretically, LAG-3/PD-1 are co-expressed on the same cell) was stronger than that of the parental antibody. Therefore, we speculated that YG-003D3 could not only relieve the inhibition of immune checkpoints but also promote the aggregation of immune cells by bridging PD-1^+^ and LAG-3^+^ cells, which was not available for parental monoclonal antibodies. finally, we hope that this study will provide favorable support for the design and downstream application of PD-1 and LAG-3 bispecific antibodies.

## Materials and methods

### Reagents

LAG-3-His protein (Cat. No.:LAG-HM131), LAG-3-Biotin protein (Cat. No.: LAG-HM431B), PD-1-His protein (Cat. No.: PD-1-HM101), PD-1-Biotin protein (Cat. No.:PD-1-HM401B), Human GM-CSF protein (Cat. No.: GSF-HE001) and Human IL-4 protein (Cat. No.: IL-4-HM001) were purchased from Kactus Biosystems; CD16a (FcgRIIIa, V176) Kit was purchased from Vazyme (Cat. No.: DD2401-01); Streptavidin-HRP was purchased from Thermo Fisher Scientific Co., Ltd. (Cat. No.: S911); CytoTell™ Blue (Cat. No.: 22251) and CytoTell™ Red 650 (Cat. No.: 22255) were purchased from AAT Bioquest; APC-labeled streptomycin (Streptavidin-APC) was purchased from Agilent corporation (Cat. No.: SA10-10); APC anti-human Ig light chain kappa (Cat. No.: V1031315), MojoSort™ Human CD14^+^ Monocytes Isolation Kit (Cat. No.: 480048), MojoSort™ Human CD4 T Cell Isolation Kit (Cat. No.: 480130) and ELISA MAX™ Standard Set Human kit IFN-γ) were purchased from Biolegend corporation (Cat. No.: 430101); Cytotoxicity Detection Kit PLUS (LDH) were purchased from Roche(Cat. No. 04744934001); Simple PlexTM Automated Immunoassays were purchased from protein simple corporation (Cat. No.: SPCKA-CS-004033); TMB color developing solution was purchased from Beijing ComWin Biotech Co.Ltd (Cat. No.: CW0050S); Coating solution: 0.1M sodium carbonate-sodium bicarbonate buffer (pH 9.6) was purchased from Sinopharm Chemical Reagent Co., Ltd. (Sodium Carbonate Cat. No.: 10018960; Sodium Bicarbonate Cat. No.: 1001891922); PBS was purchased from Servicebio corporation (Cat. No.: G4200-500ML); HiTrap Protein A HP antibody purification column was purchased from Cytiva (Cat. No.:17-0402-01) PBST (0.1% Tween 20 added to PBS); FACS solution (2% serum in PBS).

### Expression, purification, and assembly of bispecific antibody

Anti-human PD-1 antibody Fv78 and Anti-human LAG-3 antibody MIL80 were constructed by fusing two variable regions into the N-terminal of the IgG4-Fc domain and produced by the Chinese hamster ovary (CHO) cells, purified by protein A resin. YG-003D3 is a ‘knob-into-hole’ format bispecific antibody against PD-1 and LAG-3, and its variable region was derived from Fv78, MIL-80. Amino acid mutation (T^366^S; L^368^A; Y^407^V) on one side of symmetrical IgG1 Fc structure with the anti-LAG-3 variable region. the mutant forms a structure with a ‘groove’, that is, a hole for bispecific antibodies. Amino acid mutation (T^366^W) on the other side of symmetrical IgG1 Fc structure with the anti-PD-1 variable region. Tryptophan as replaced amino acids will form a larger spatial structure, that is, the bispecific antibody Knob. At the same time, to eliminate the ADCC effect, we mutated the antibody’s heavy chain amino acid(N^297^A). Briefly, half antibodies were expressed in separate CHO cell cultures. After culturing for 7 days, the supernatant was collected and purified by protein A affinity chromatography column. The purified Knob and Hole were mixed according to the molar ratio of 1:1, and then the protein content was diluted to 5.5 g/L. In the presence of Glutathione (GSH), adjust the mixed solution to pH8.0 and stirred overnight at low speed (24 hours) at room temperature. After the reaction, the pH was adjusted to 5.5 with 2M acetic acid. The formation of heavy chain heterodimers was promoted in the presence of a reductant, and the disulfide bonds between light and heavy chains would not be opened under this condition in theory, a 50KDa ultrafiltration tube was used to remove the free reductant to obtain the bispecific antibody after assembly. Then, Size Exclusion Chromatography(SEC, which mainly analyzed the proportion of haptens) and Hydrophobic interaction chromatography(HIC, which mainly analyzed the proportion of homologous dimers and heterodimers) were used to analyze the specific components in the assembled samples.

### Cell culture and engineering cell construction

293T, Raji, and MC38 cells were from the American Type Culture Collection Center (ATCC). 293T and MC38 cells were cultured in the DMEM medium containing 10% FBS. Raji cells were cultured in RPMI 1640 medium containing 10% FBS. The above cells were placed in an incubator at constant temperature and humidity at 37 degrees Celsius with 5% CO2. All cells were cultured for more than three generations, and the cells were in the logarithmic growth phase before the experiment.

CHO-K1 cells were purchased from the ATCC and transfected with plasmids to stably express human PD-L1(CHO-K1(hPD-L1) for short). 293-T cells were transfected with two plasmids to stably express human LAG-3 and PD-1(293T(PD-L1/LAG-3) for short)). The human PD-L1 knock-in MC38 cell line (Mc38(hPD-L1) for short) was constructed by Shanghai Model Organisms Center Co., Ltd. Briefly, mouse PD-L1 was replaced by human PD-L1 expression frame based on CRISPR/Cas9 technology.

### Mice

All animal experiments were performed in accordance with regulations for the care and use of laboratory animals at the Specific Pathogenic Free (SPF) animal house and were approved by the Institutional Animal Care and Use Committee. Specifically, Human PD-1/LAG-3 double knock-in mice were purchased from Shanghai Model Organisms Center (strain: C57BL/6-Pdcd1^tm1(PDCD1^) LAG-3^tm1(LAG-3)^/Bcgen). All mice were maintained under specific pathogen-free conditions.

### HTRF binding assay to test the binding activity of YG-003D3 to CD16a

According to the instructions of CD16a (FcgRIIIa, V176) Kit, 5 μL of bispecific antibody YG-003D3 was first added to the 384 shallow hole plate (starting at 200 μg/mL, 3-fold dilution, six gradients), and the parental antibody control and positive control (Herceptin) were set. Then, 5 μL diluted IgG-A2, Tag1-CD16a (V176), and Anti-Tag1-Eu were added respectively, and they were gently mixed in the injection hole with a liquid shifter. After incubation at room temperature for 2 hours, the emission light at two wavelengths (665 nm and 620 nm) was detected by a microplate reader (equipped with HTRF/TR-FRET module). The fluorescence value at 665 nm was divided by the fluorescence value at 620 nm to obtain the 665/620 value. The smaller the ratio, the stronger the binding ability of the antibody to CD16a.

### BLI analysis of antibody affinity

BLI technology was used to detect antibody affinity. The YG-003D3/MIL-80/Fv78 were fixed on the Anti-Human IgG Fc Capture (AHC) chip, and the PD-1-His or LAG-3-His proteins were serially diluted with protein interaction running buffer solution (starting at 50nM, 2-fold dilution, 7 gradients). The association reaction time was set at 180s, the dissociation reaction time was set at 180s. After the reaction, the reaction was regenerated in 10 mM glycine HCl (pH = 1.7) for 5 seconds, and in protein interaction running buffer solution for 5 seconds. The regeneration-infiltration step was repeated 3 times. Data analysis 7.0 software was used to analyze the affinity of antibody samples.

### BLI analysis of the binding activity of YG-003D3 to PD-1/LAG-3 proteins simultaneously

To evaluate the ability of YG-003D3 to bind PD-1 and LAG-3 simultaneously, YG-003D3 (100nM) was captured by the AHC chip, and then LAG-3-his (50nM) protein was binding as the first antigen. when YG-003D3 was saturated with LAG-3, PD-1-his (starting at 100nM, 4-fold dilution, 3 gradients) protein was the second antigen to test the binding activity of PD-1 to YG-003D3. During this period, the first antigen was set as blank and the second antigen was set PD-1 of the same concentration as the control, to compare whether the presence or absence of LAG-3 would affect the binding activity of PD-1 to YG-003D3.

### Double-antigen sandwich ELISA assay to test the binding activity of YG-003D3 to PD-1/LAG-3 proteins simultaneously

LAG-3-His or PD-1-His (1μg/mL) protein was coated overnight at 4°C as a stationary phase. On the second day, the unknotted protein was washed with PBST, and 4% milk was blocked at 37°C for an hour. After the closure, the milk was washed, and the bispecific antibody YG-003D3 was diluted to the corresponding concentration (starting at 50nM, 2-fold dilution, 12 gradients). At the same time, the parental antibody was set as the control. The reaction was carried out at 37°C for an hour, and the unconjugated product was washed. The unconjugated product was incubated with 2 μg/mL PD-1-Biotin or LAG-3-Biotin for 1 hour, and then the unconjugated product was washed. After incubation with Streptavidin-HRP for 0.5 hours, TMB was used for color development. The reaction was terminated by 1 M sulfuric acid solution, and the absorbance at OD_450_ nm was measured by an enzyme-labeled instrument.

### Cell-based binding assay

The binding activity of antibodies to target-expressing cells was assessed using 293T(PD-1/LAG-3) and human PBMCs (PBMCs were isolated from the whole blood of healthy donors using human lymphocyte separation solution). First, YG-003D3, FV78, MIL-80, and IgG1 antibodies were diluted with FACS solution (starting at 100nM, 3-fold dilution), incubated with the above cells at 4°C for 30 min, and then washed with FACS and stained with an APC anti-human Ig kappa light chain for 30 min at 4°C. Thereafter, cells were washed and suspended in 1% paraformaldehyde for flow cytometry analysis, and the positive rate was calculated.

### Validation of blocking effect of YG-003D3 on the binding of PD-1 to its ligand by competitive ELISA

PD-L1 or PD-L2 protein was coated at 4°C overnight; on the second day, the unknotted proteins were washed with PBST. After blocking with 4% milk at 37°C for an hour, the milk was washed away, and the bispecific antibody YG-003D3 was diluted with 5μg/mL PD-1-Biotin to the corresponding concentration (starting at 100 nM, 2-fold dilution, 12 gradients), and added to the orifice plate. Meanwhile, the parents were set in control. After incubation at 37°C for 1 hour, the unconjugated samples were washed away, and finally incubated with Streptavidin-HRP for 0.5 hours, TMB was used for color development. The reaction was terminated by 1M sulfuric acid solution, and the absorbance at OD_450_ nm was measured by a microplate reader.

### Cell-based blocking assays

YG-003D3 was diluted with PD-1-Biotin protein at a concentration of 5μg/mL to the corresponding concentration (starting at 200nM, 2-fold dilution, 8 gradients), and incubated with CHO-K1(hPD-L1) at 4°C for 30 min. Cells were washed with FACS and stained with Streptavidin-APC for 30 min at 4°C. Thereafter, cells were washed and suspended in 1% paraformaldehyde for flow cytometry analysis, and a positive rate was calculated. YG-003D3 was diluted with LAG-3-Biotin protein at a concentration of 5μg/mL to the corresponding concentration (starting at 200nM, 2-fold dilution, 8 gradients), and incubated with Raji cells expressing human MHC-II at 4°C for 30 min. Cells were washed with FACS and stained with Streptavidin-APC for 30 min at 4°C. Thereafter, cells were washed and suspended in 1% paraformaldehyde for flow cytometry analysis, and a positive rate was calculated.

### Crosslinking of PD-1^+^ cells with LAG-3^+^ cells

CytoTell™ Blue-labeled PD-1-expressing 293T cells and CytoTell™ Red 650-labeled LAG-3-expressing 293-T cells were mixed 1:1 and incubated with YG-003D3 (100nM), Fv78 (100nM), MIL-80 (100nM), or an IgG1 (100nM) control, double positive events were detected by flow cytometry.

### PBMCs activation experiment

PBMCs activation experiment was used to evaluate the activation effect of bispecific antibodies. PBMCs were resuspended with the 1640 culture medium containing Human CD3/CD28 antibody (0.1μg/mL) and placed in a 96-well plate (1.25×10^5^ cells per well). Add YG-003D3 antibody (set three concentration gradients, 100nM, 10nM and 1nM) to stimulate. After three days, the expression of IFN-γ in PBMCs was detected by ELISA. At the same time, the parental control group, Fv78/MIL-80 group (set three concentration gradients according to the same number of moles of antigen binding domain as the bispecific antibody, 50nM, 5nM, and 0.5nM) and parental combination group (both Fv78 and MIL-80 is added at the same concentration as the following gradients: 50nM, 5nM, 0.5nM, respectively) were established. The supernatants of PBMCs stimulated with the first two antibody concentrations were selected for each group, and the expression levels of IL-2, IL-6, and TNF-α in the supernatant were detected by Simple PlexTM Automated Immunoassays.

### Mixed lymphocyte reactions

Isolation of mononuclear cells from PBMCs by MojoSort™ Human CD14^+^ Monocytes Isolation Kit. The cells were resuspended in 10% FBS 1640 medium and counted. The cells were inoculated in a 96-well plate at a rate of 2×10^4^/well. The monocytes were stimulated to differentiate into DC cells by 50 ng/mL Human-IL-4 and 25 ng/mL Human-GM-CSF. The cells were cultured in a 37°C cell incubator for 7 days, and the solution was changed every three days. On the 7th day of culture, PBMCs were isolated from the whole blood of another healthy volunteer by the same method. CD4^+^ T positive cells were sorted by a CD4^+^ T cell magnetic bead sorting kit. The cells were resuspended in 10% FBS 1640 medium and counted. The cells were seeded in 96-well plates at 2×105/well. At the same time, YG-003D3, Fv78, or MIL-80 antibody was diluted to the appropriate concentration in the 10% FBS 1640 medium. The gradient diluted antibody was added to the 96-well plate with CD4^+^ T cells. The supernatant was harvested after being cultured in a 37°C cell incubator for 5 days, and the expression of IFN-γ in the supernatant was determined using a human IFN-γ ELISA kit.

### Killing effect of PBMCs on tumor cells *in vitro*

MC38-PD-L1 cells and PBMC were mixed in a ratio of 1:10 in 96-well plates, that is, 2×10^4^ tumor cells mixed with 2×10^5^ isolated PBMC per well. At the same time, YG-003D3 antibody was diluted to an appropriate concentration (starting at 400nM, 5-fold dilution, 3 gradients) in MEM medium. Gradient diluted antibodies are added to the above 96-well plates. After 6 hours of culture in 37 C cell incubator, the supernatant was collected and the killing effect of PBMC on tumor cells was detected by Cytotoxicity Detection Kit PLUS (LDH). At the same time, the parental control group, Fv78 or MIL-80 group (set three concentration gradients according to the same number of moles of antigen binding domain as the bispecific antibody, 200nM, 40nM, and 8nM) and parental combination group (both Fv78 and MIL-80 is added at the same concentration as the following gradients: 200nM, 40nM, 8nM, respectively) were established.

### Tumor models

Human PD-L1 knock-in MC38 cells (1.5×10^6^) were implanted subcutaneously into the right flank of human PD-1/LAG-3 double knock-in female mice. On day 6 post-tumor implantation, mice were randomized into four groups (n=6 in each group) with a mean tumor volume of approximately 80-120 mm^3^. On days 0, 3, 7, 10 and 14 post grouping, mice were intraperitoneally (i.p.) with YG-003D3 (2mg/kg), FV78+MIL-80 (1mg/kg+1mg/kg), YG-003D3 (6mg/kg), and isotype IgG (6mg/kg). Tumor volume and body weight were measured twice a week. Tumor volume was calculated using the formula: (length×width^2^)/2. Mice were euthanized when tumor volume reached 2000 mm^3^. In terms of the potential hematological toxicity, the mice’s blood was collected 24 hours before and after the fourth administration for the routine blood test and blood biochemical test after the last administration. Blood routine test indicators include WBC, RBC, HGB, HCT, MCV, MCH, MCHC, PLT, RDW-SD, RDW-CV, PDW, MPV, P-LCR, PCT, NEUT^#^, LYMPH, MONO^#^, EO^#^, BASO^#^, NEUT%, LYMPH%, MONO%, EO%, and BASO%. Blood biochemical test indicators include alanine aminotransferase (ALT), aspartate aminotransferase (AST), blood urea nitrogen (BUN), and creatinine (CRE). Blood and tumor tissues were collected at the end of the experiment. The contents of CD45^+^ cells, CD3^+^ T cells (CD45^+^/CD3^+^), CD4^+^ T cells (CD45^+^/CD3^+^/CD4^+^), CD8^+^ T cells (CD45^+^/CD3^+^/CD8^+^) and Regulatory T cells (Treg, CD45^+^/CD3^+^/CD4^+^/CD25^+^/Foxp3^+^) in blood and tumor tissues were labeled with specific antibodies and detected by flow cytometry to observe the rate of T cells in blood and tumor tissues.

In order to fully study the anti-tumor advantages of BsAb, we conducted a set of experiments later. The tumor therapeutic effects of YG-003D3 (4mg/kg) and FV78+MIL-80 (2mg/kg+2mg/kg) on tumor-bearing mice were compared. MC38 (hPD-L1) cells (1.5×10^6^) were implanted subcutaneously into the right flank of human PD-1/LAG-3 double knock-in female mice. On day 6 post-tumor implantation, mice were randomized into two groups (n=6 in each group) with a mean tumor volume of approximately 80-120 mm^3^. On days 0, 3, 7 and 10 post grouping, mice were intraperitoneally with YG-003D3 (4mg/kg) or FV78+MIL-80 (2mg/kg+2mg/kg). Tumor volume and body weight were measured. Tumor volume was calculated using the formula: (length×width^2^)/2. During the administration, the tumor size and the weight of the mice were measured, after stopping the treatment, the tumor volume and tumor weight were observed until the end of the experiment. At the end point, the dissected tumors were weighed, photographed, and statistically analyzed.

### Statistical analyses

Statistical analyses were performed using GraphPad Prism 6.0 statistical software (GraphPad Software Inc). All *in vitro* experiments were replicated at least 3 times under the same experimental conditions. EC50 values were calculated using nonlinear regression analysis. The statistical significance of tumor volume and tumor weight between groups at the end of treatment was determined by One way ANOVA/Two way ANOVA with the Tukey test. Unpaired t test was used to compare the statistical significance of changes in the proportion of immune cells in tumor and peripheral blood of mice. Dates were considered statistically significant when p values lower than 0.05 (*p <0.05, **p < 0.01, ***p<0.001 and ****p <0.0001).

## Results

### Preparation of YG-003D3, a BsAb targeting human PD-1, and LAG-3

**Figure 1:**
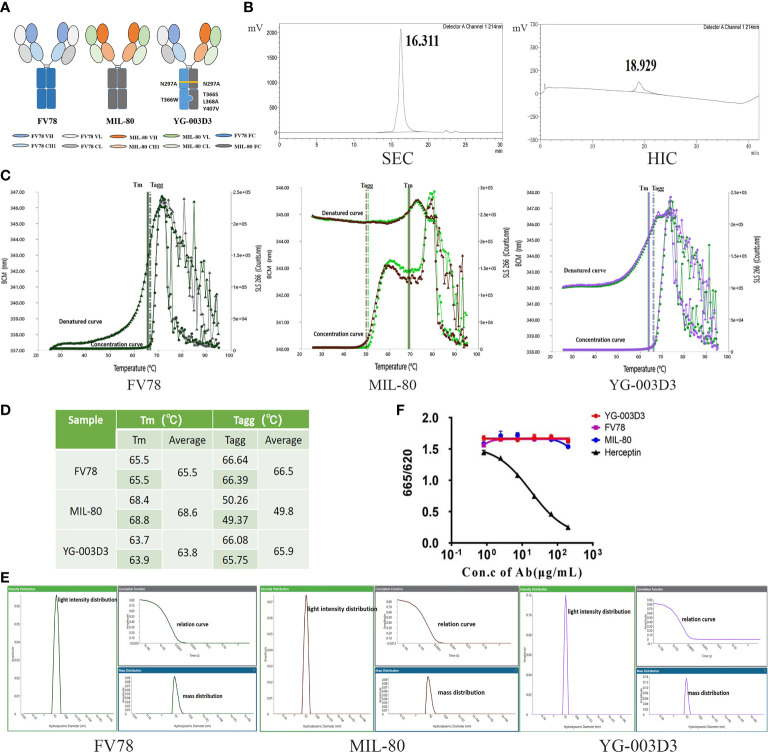
*In vitro* assembly and quality analysis of YG-003D3. **(A)** Schematic structure of YG-003D3, Fv78, and MIL-80. Among them, YG-003D3 involves the engineering of Fc, mainly including Fc mutation T^366^S; L^368^A; Y^407^V for the hole with the anti-LAG-3 variable region and T^366^W for the knob with the anti-PD-1 variable region. The structure of ‘knob’ and ‘hole’ promotes the formation of heterodimers in the heavy chain. The N^297^A mutation eliminates Fc-mediated cross-linking of target cells and immune cells. **(B)** SEC-HPLC results confirmed that the assembled antibody did not contain semi-antibody and some small fragment components. Since the hydrophilicity and hydrophobicity of Knob and Hole proteins were different, HIC-HPLC was used to further analyze whether there was a Knob/Hole homodimer in the assembled antibody. **(C, D)** The denaturation temperature (Tm) and aggregation temperature (Tagg) of the bispecific antibody and two parental antibodies and YG-003D3 were detected. **(E)** Mass and light intensity distribution of YG-003D3, Fv78, and MIL-80, which suggests that the YG-003D3 antibody was homogeneous. **(F)** The binding activity of BsAb YG-003D3 to human CD16a.

According to the strategy for the construction of BsAb in ‘knob-into-hole’ structures described in method 2.2. **Figure 1A** delineates the structural characteristics of parental antibodies and BsAb, YG003-D3 forms a heterodimer through the engineered mutation of Fc, and it is obvious that the structure of this bispecific antibody is very similar to that of natural antibody. The purified semi-antibody components were analyzed by SDS-PAGE and SEC (**Supplementary Figure 1**). Then, the purified Knob and Hole were mixed under the condition of GSH reduction to promote the formation of heterodimer bispecific antibody. SEC results confirmed that the assembled antibody contains only one main peak and almost contain no semi-antibody and small fragment components (**Figure 1B**). However, Knob/Hole homodimers cannot be excluded from the SEC detection. Due to the differences in the hydrophobicity of Knob and Hole proteins, we used HIC to further analyze the assembled BsAb. The results showed that the main peak time of the Knob was 19.876 min, and the peak time of the Hole was 16.106 min and 25.041 min respectively (Hole prone to self-polymerization). However, there was only one peak time (18.929 min) detected in the assembled BsAb, which confirmed that the correctly assembled BsAb YG-003D3 was nearly 100%. To test the initial stability of the bispecific antibody, we measured the stability and light intensity distribution of YG-003D3. The results showed that the average denaturation temperature (Tm) and aggregation temperature (Tagg) of YG-003D3 were 63.8°C and 65.9°C respectively. Comparatively, the average Tm/Tagg value of Fv78 was both at 65.5°C, and the average Tm/Tagg value of MIL-80 was 68.6°C and 49.8°C. The above results showed that YG-003D3 had good structural stability (**Figures 1C, D**). The mass light intensity distribution results also showed that the YG-003D3 has high purity with a single component (**Figure 1E**). Finally, to determine whether the N297A mutation was effective in eliminating the potential ADCC effect of the bispecific antibody, we tested the binding activity of the YG-003D3 to human CD16a using the HTRF assay. The results are shown in **Figure 1F**. YG-003D3 cannot bind to CD16a after IgG1(N297A) mutation. In addition, MIL-80 and Fv78 belong to the IgG4 subtype and also do not bind to CD16a, In contrast, the positive control Herceptin could effectively bind CD16a in a dose-dependent manner.

### The binding properties of YG-003D3

**Figure 2:**
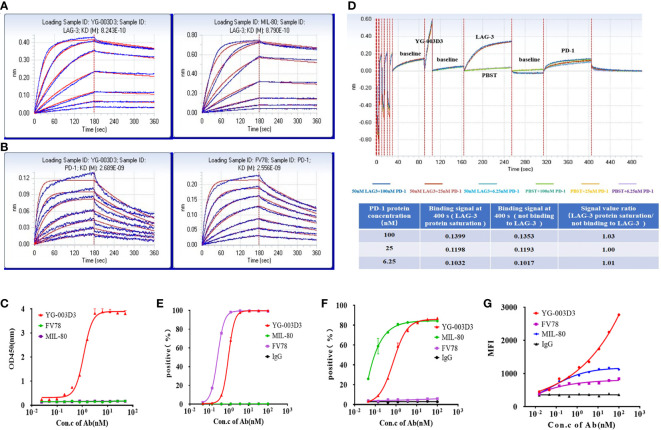
The binding activity of YG-003D3 to human PD-1 and LAG-3. **(A)** BLI Verification of affinity of YG-03D3 or MIL-80 to LAG-3. The affinity of YG-003D3 to LAG-3 was 824.3 pM, which was similar to that of MIL-80 (879.0 pM). **(B)** BLI Verification of affinity of YG-03D3 or Fv78 to PD-1. The affinity of YG-003D3 to PD-1 was 2689.0 pM, which was similar to that of Fv78(2556.0 pM). **(C)** Double-antigen sandwich ELISA verified the bispecific antibody could recognize LAG-3 and PD-1 simultaneously. **(D)** BLI verified that there was no steric hindrance in the binding of the bispecific antibody to LAG-3 and PD-1 simultaneously. **(E)** YG-003D3 binds to PD-1-expressing cells in a dose-dependent manner. **(F)** YG-003D3 binds to LAG-3-expressing cells in a dose-dependent manner. **(G)** YG-003D3 binds to activated PBMCs in a dose-dependent manner, moreover, YG-003D3 has a better binding ability to activated PBMCs than parental antibodies. All experiments were repeated three times.

We evaluated the affinity of YG-003D3, MIL-80, and Fv78 to human PD-1 and LAG-3 by BLI. As shown in **Figure 2**, the affinity of YG-003D3 to LAG-3 was 824.3 pM, which was similar to that of MIL-80 (879.0 pM, **Figure 2A**). The affinity of YG-003D3 to PD-1 was 2689.0 pM, which was similar to that of Fv78 (2556.0 pM, **Figure 2B**). The results showed that the BsAb did not change the affinity of the two antibody binding sites to their respective antigens. Double-antigen sandwich ELISA were used to verify that the bispecific antibody could simultaneously target two targets of LAG-3/PD-1. As expected, YG-03D3 binds to both human PD-1 and LAG-3, while neither Fv78 nor MIL80 as parental antibodies could not bind to both antigens simultaneously (**Figure 2C**). Similar results were obtained by changing the position of the two sandwich antigens (**Supplementary Figure 2**). Meanwhile, BLI technology was used to further verify whether there was a spatial steric hindrance of YG-003D3 in the process of recognizing PD-1/LAG-3 simultaneous. The results are shown in **Figure 2D**. Whether YG-003D3 binds to LAG-3 protein or not, it does not affect its binding signals to PD-1 protein, the binding signal ratio (When two binding sites do not affect each other, their binding signal intensities should be similar.) is about 1 at 3 sets of PD-1 concentrations. This result shows that YG-003G3 is independent of each other in the process of binding to the two antigens. To confirm the binding ability of YG-003D3 to the cell surface of PD-1 and LAG-3 proteins, flow cytometry was performed in target-overexpressing cell lines and PBMCs. YG-003D3 showed dose-dependent binding to human PD-1-expressing 293T cells(**Supplementary Figure 3A**), LAG-3-expressing 293T cells(**Supplementary Figure 3B**), and PBMCs (**Figures 2E-G**). Taken together, these results confirmed that in cells expressing PD-1/LAG-3 alone (293T-PD-1^+^/293T-LAG-3^+^), the binding of YG-003D3 to PD-1 or LAG-3 was weaker than that of the corresponding parent antibody (Fv78/MIL-80), which may be caused by the reason that the monoclonal antibody has two identical antigen-binding regions for membrane antigen binding, however, the bispecific antibody only has one antigen binding region for the same antigen. Therefore, we used PD-1 and LAG-3 double-expressed activated PBMCs (**Supplementary Figure 3C**) to test the binding activity of YG-003D3 to both antigens. As shown in **Figure 2G**, YG-003D3 has a stronger binding ability to PBMCs than parental antibodies When PD-1/LAG-3 were simultaneously expressed on the same cell surface.

### The blocking properties of YG-003D3

**Figure 3:**
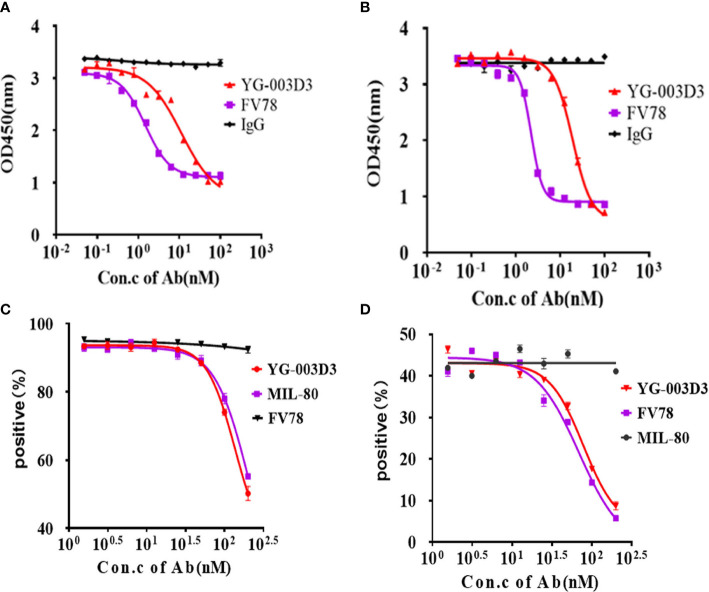
YG-003D3 blocks the interaction between PD-1/PD-L1, PD-1/PD-L2, and LAG-3/MHC-II. **(A)** Competitive ELISA assay to test the blocking activity of YG-003D3 on the interaction of PD-1/PD-L1. **(B)** Competitive ELISA assay to test the blocking activity of YG-003D3 on the interaction of PD-1/PD-L2. **(C)** Flow cytometry to test blocking activity of YG-003D3 on binding of LAG-3-biotin to Raji cells (Constitutive expression of MHCII). **(D)** Flow cytometry to test the blocking activity of YG-003D3 on binding of PD-1-biotin to PD-L1-overexpressing CHO cells (CHO-K1(PD-L1). All experiments were repeated three times.

The BsAb YG-003D3 was designed to restore the function of depleted T cells for cancer treatment by blocking PD-1/LAG-3 immune checkpoint on immune cells, especially those depleted T cells. Therefore, it is essential to verify the blocking effect of YG-003D3 on PD-1/PD-L1, PD-1/PD-L2, and LAG-3/MHC-II. Firstly, competitive ELISA was used to verify the blocking effect of YG-003D3 on PD-1/PD-L1 and PD-1/PD-L2 *in vitro*. The results confirmed that YG-003D3 could block the interaction of PD-1/PD-L1 and PD-1/PD-L2 in a dose-dependent manner (**Figures 3A, B**). It can be seen from the results that the blocking ability of YG-003D3 to PD-1/PD-L1 and PD-1/PD-L2 binding was weaker than that of the parental antibody. Well, the likely reason is that YG-003D3 had only one PD-1 binding arm while Fv78 had two. Next, Raji cells expressing MHC-II(**Supplementary Figure 3D**) and CHO-K1(hPD-L1) cells expressing human PD-L1(**Supplementary Figure 3E**) were used to verify the blocking activity of antibodies at the cellular level. As results were shown in **Figure 3C** and **Figure 3D**. The bispecific antibody YG-003D3 can block the binding of LAG-3/MHC-II and PD-1/PD-L1 in a dose-dependent manner at the cellular level. The blocking ability of YG-003D3 on LAG-3/MHC-II or PD-1/PD-L1 is similar to that of its parent antibody MIL-80 or FV78.

### *In vitro* functional study of YG-003D3

**Figure 4:**
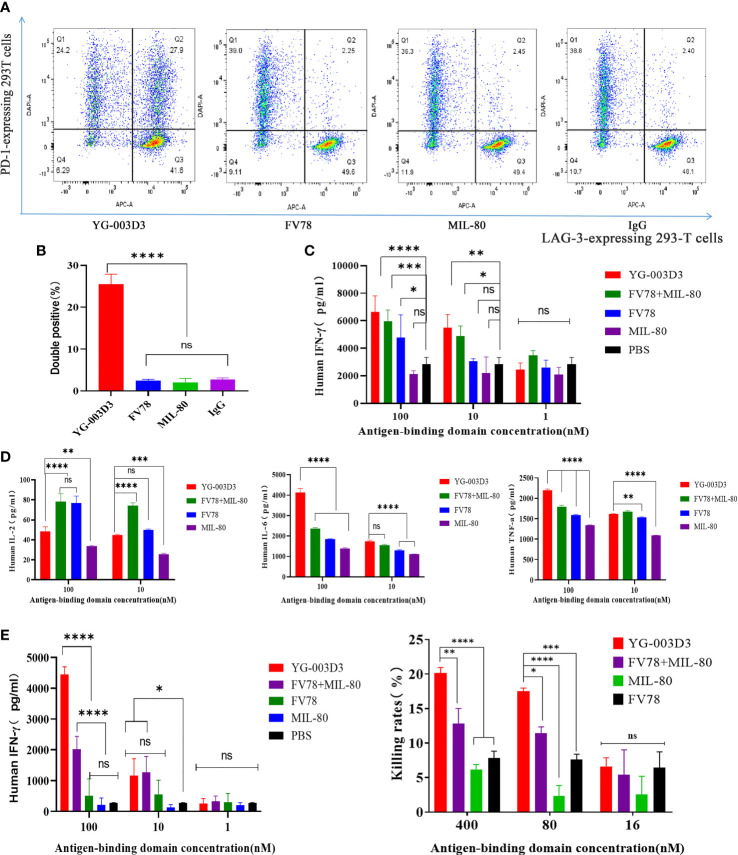
*In vitro* biological activity of YG-003D3. **(A)** CytoTell™ Blue-labeled PD-1-expressing 293T cells and CytoTell™ Red 650-labeled LAG-3-expressing 293-T cells were mixed 1:1, then, flow cytometry scatter diagram showing the percentage of dual positive cells treated with YG-003D3, Fv78, MIL-80, respectively. YG-003D3 could have a marked increase in the crosslinking ratio of PD-1/LAG-3 dual positive cells when compared with other groups. **(B)** YG-003D3, Fv78, MIL-80, and control antibody IgG group double positive cell complex ratio statistics, data represent three independent experiments. **(C, D)**. YG-003D3 double blocking PD-L1/LAG-3 pathway can promote PBMCs activation. It can be found from the figure that when the concentration of the same antigen binding region was the same, YG-003D3 could promote PBMCs to secrete more IFN-γ, IL-6, and TNF-α than the parental antibody. Although the promotion effect of YG-003D3 on IL-2 secretion by PBMCs was not obvious, the overall activation effect of YG-003D3 on PBMCs was significantly better than that of Fv78 and MIL-80 monoclonal antibodies. **(E)** YG-003D3 enhances CD4^+^ T cell activation. IFN-γ secretion in culture was measured after co-culturing CD4^+^ T cells with monocyte-derived DCs for 5 days with YG-003D3, Fv78, MIL-80, or Fv78+MIL-80. The results showed that a high concentration of bispecific antibody YG-003D3 had a strong effect on promoting T cell activation. F.YG-003D3 promotes PBMCs to kill tumor cells. Data are derived from human PBMCs from 3 healthy donors. All experiments were repeated two or three times. Dates were considered statistically significant when p values lower than 0.05 (*p <0.05, **p < 0.01, ***p<0.001 and ****p <0.0001)..

Flow cytometry was used to further assess the binding potential of YG-003D3 to PD-1 and LAG-3 molecules on two different cells simultaneously. CytoTell™ Blue-labeled PD-1-expressing 293T cells and CytoTell™ Red 650-labeled LAG-3-expressing 293-T cells were mixed and incubated with YG-003D3, Fv78, MIL-80, or an IgG1 control under specific concentration, double positive events were detected by flow cytometry. The results showed that YG-003D3 significantly increased the cross-linking of PD-1**^+^** cells and LAG-3**^+^** cells, and the proportion of double-positive events was up to 27.9%. While Fv78, MIL-80, and IgG antibodies don’t cause cross-linking of the two cells, the proportion of double-positive events was 2.25%, 2.45%, and 2.4% (**Figure 4A**). The experiments were repeated three times, the statistical results of double positive ratio in each group are shown in **Figure 4B**, it’s obvious that YG-003D3 can significantly promote the crosslinking of two cells. Next, we verified whether the bispecific antibody YG-003D3 could activate PBMCs to release IFN-γ at the cellular level. As shown in **Figure 4C**, YG-003D3 could significantly activate the PBMCs, even better than Fv78, MIL-80 treated alone when antigen binding region concentration is the same. Another interesting point is that MIL-80 alone can hardly promote the release of IFN-γ from PBMCs, but when combined with Fv78, it will enhance the activation ability of Fv78 on PBMCs, and the activation intensity is similar to that of bispecific antibody YG-003D3. However, the activation effect of low-concentration antibodies on PBMCs was not obvious, other cytokines are also not well detected in this method, so we used Simple Plex™ Automated Immunoassays to analyze the release of IL-2, IL-6, and TNF-α by PBMCs stimulated by high-concentration bispecific antibody YG-003D3. The result was shown in **Figure 4D**. Compared with the parental antibody group, YG-003D3 could significantly stimulate PBMCs to secrete IL-6 and TNF-α, and its stimulation effect was better than MIL-80+Fv78 combination. Although the ability of YG-003D3 to stimulate PBMCs to secrete IL-2 was weaker than that of the Fv78 group and parental antibody combination group. In general, the activation effect of YG-003D3 on PBMCs was significantly better than that of Fv78 and MIL-80 monoclonal antibodies. We used Mixed Lymphocyte Reaction (MLR) to further verify the T cell activation of YG-003D3. As shown in **Figure 4E**, a high concentration of YG-003D3 can strongly stimulate T cells to express IFN-γ, and the parental control group (Fv78/MIL-80) almost did not detect the increase in IFN-γ expression obviously. Although, There was a dose dependence in the combination group. However, at high concentrations(100nM), YG-003D3 had a significant effect on IFN- γ, the activation and release ability was significantly better than that of single drug group and combined drug group. These results suggest that both bispecific antibody and combination therapy has better T cell activation advantage than a parental monoclonal antibody. Finally, we verified whether YG-003D3 can promote the killing effect of PBMCs on tumor cells *in vitro*. As shown in **Figure 4F**, the killing rate of PBMCs on tumor cells MC38 was as high as 20% under the stimulation of high dose YG-003D3 (400nM). Whether YG-003D3 in the high dose group(400nM) or the middle dose group(80nM), the killing effect of the induced immune cells on tumors is significantly stronger than that of the single drug group under the same dose(P<0.001, Two way ANOVA), and even better than that of the combined group under the same dose(P<0.05, Two way ANOVA). In conclusion, compared with the combined group or the monoclonal antibody group, YG-003D3 has a stronger tumor killing effect *in vitro*.

### Anti-tumor efficacy of YG-003D3 in humanized mouse models

**Figure 5:**
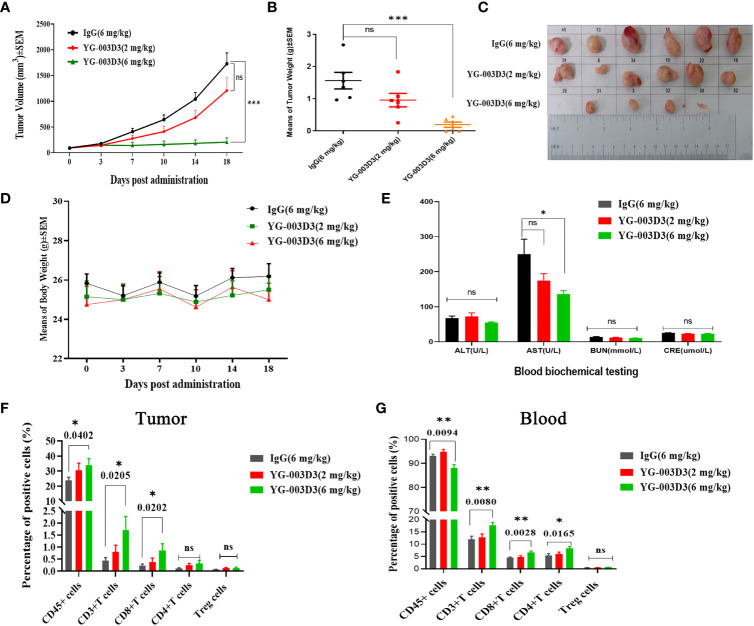
Anti-tumor effect of YG-003D3 in the humanized mouse model. **(A)** Tumor growth trend chart in human PD-1/LAG-3 double knock-in mice bearing MC38(hPD-L1) were treated with antibodies. **(B)** At the end of the experiment, animals were euthanized, and tumor blocks were stripped and weighed. The tumor weight of the IgG control group was 1.5633 ± 0.2560g, and the tumor weight of the median-dose YG-003 D3 group was 0.1954 ± 0.0815g. The tumor weight inhibition rate (IR) was as high as 87.50%, which was significantly different from the tumor volume of the control group (P < 0.001). **(C)** Inspection of tumor tissues excised from each group at the end of the study. **(D)** Mouse body weight changes during antibody drug therapy. **(E)** Blood biochemistry data after the experiment. **(F)** Detection of the proportion of immune cells of various class in tumor tissues. **(G)** Detection of the proportion of immune cells of various class in the peripheral blood. The results demonstrate that YG-003D3 can induce an increase in the proportion of T cells exerting a tumor-killing effect in tumors and peripheral blood, and exert an excellent antitumor effect. Dates were considered statistically significant when p values lower than 0.05 (*p <0.05, **p < 0.01 and ***p<0.001).

**Figure 6:**
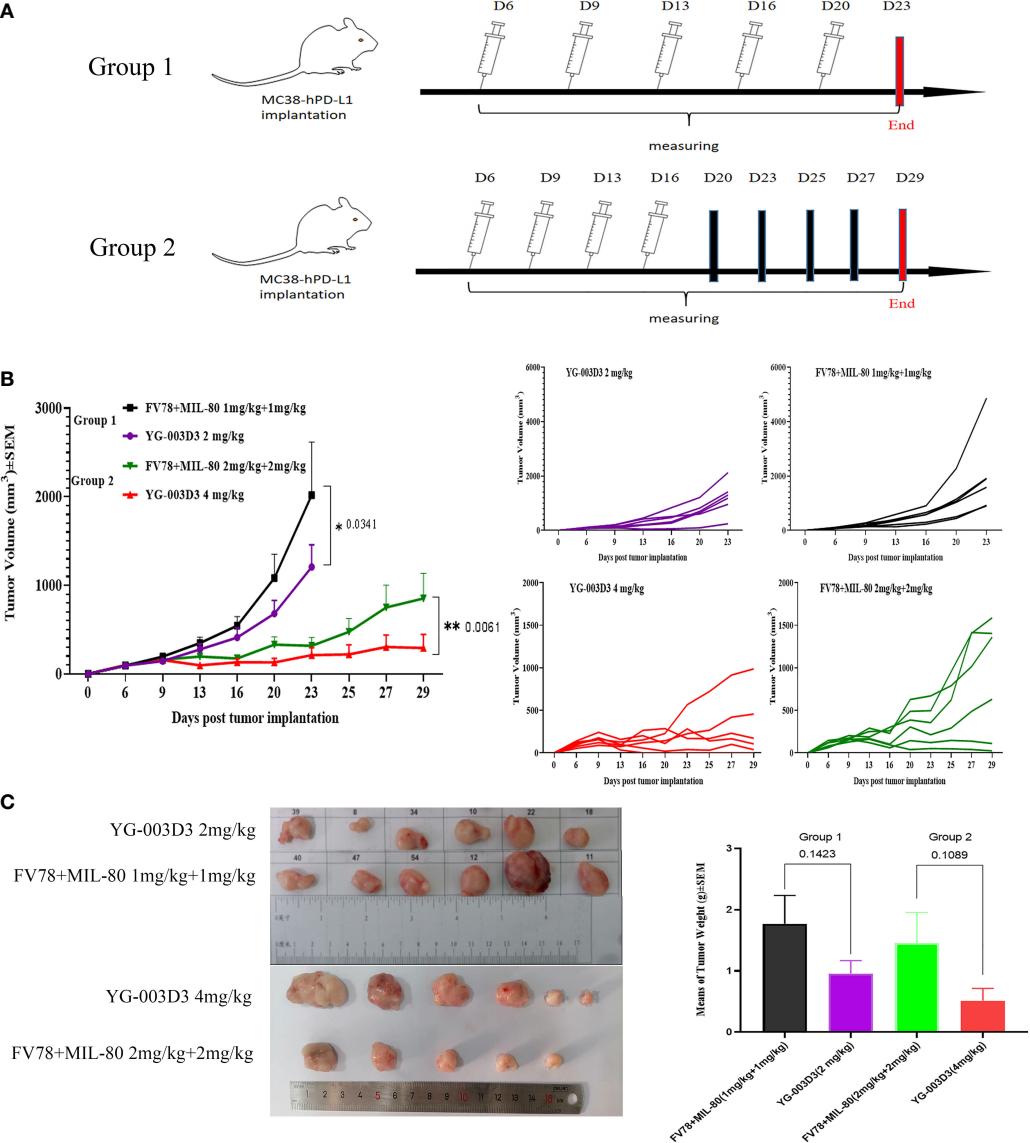
Comparison of antitumor effect between YG-003D3 and combination group. **(A)** The schedule in human PD-1/LAG-3 double knock-in mice bearing MC38(hPD-L1) were treated with antibodies (n=6 mice/group). **(B)** Tumor growth trend chart in human PD-1/LAG-3 double knock-in mice bearing MC38(hPD-L1) were treated with antibodies. **(C)** At the end of the experiment, animals were euthanized, and tumor blocks were stripped and weighed. Dates were considered statistically significant when p values lower than 0.05 (*p <0.05, **p < 0.01).

Firstly, to clarify the anti-tumor effect of YG-003D3, human PD-1/LAG-3 double knock-in mice Subcutaneous inoculated human PD-L1 knock-in MC38 colon carcinoma cell lines were used. On the sixth day after inoculation, the average tumor volume was about 94 mm^3^. The tumor-bearing mice were randomly divided into four groups, including the low-dose YG-003D3 group (2 mg/kg), FV78+MIL-80(1mg/kg+1mg/kg) groups, median-dose YG-003D3 group (6 mg/kg) and IgG control group (FV78+MIL-80(1mg/kg+1mg/kg) groups will be discussed specifically in **Figure 6**). As shown in **Figure 5A**, YG-003D3 inhibited tumor growth in a dose-dependent manner. On the 18th day after drug administration, the tumor volume in the IgG control group was 1728.74 ± 210.29mm^3^. The tumor volume of the median-dose YG-003D3 group was 205.66 ± 84.55mm^3^, and the tumor inhibition rate (TGI) was 93.19%, which has obvious statistical significance from that of the control group (P < 0.001). The tumor volume of the low-dose YG-003D3 group was 1207.64 ± 250.61mm^3^, and the TGI was 31.88%. At the end of the experiment, animals were given euthanasia, and the tumor were stripped. Further analysis of tumor weight was shown in **Figure 5B**, the tumor weight of the IgG control group was 1.5633 ± 0.2560g, and the median-dose YG-003D3 group was 0.1954 ± 0.0815g. The tumor weight inhibition rate (IR) was as high as 87.50%, which was significantly different from that of the IgG control group (P < 0.001). The low-dose YG-003D3 group was 0.9576 ± 0.2100g, and the tumor weight IR was 38.74%. It can be found that YG-003D3 treated mice showed a significant reduction in tumor volume and tumor weight compared to the IgG group. It is worth noting that in the medium-dose YG-003D3 group, the tumors of 2 mice completely disappeared (one mouse completely disappeared on the 10th day after the start of treatment, and one mouse completely disappeared on the 14th day after the start of treatment). One mouse left only 0.0587 g of tumor on the 18th day after the start of treatment, and the tumor elimination rate reached 33.33%. No tumor regression was observed in the IgG control group and the low-dose YG-003D3 group(**Figure 5C**). In addition, in order to study the preliminary safety of the YG-003D3, **Figure 5D** shows that there were no significant weight loss occurred in the 3 groups of mice during treatment, indicating that drug did not cause significant toxic side effects. To further evaluate the potential hematological toxicity and anti-tumor effect of YG-003D3 in more detail, more tests have been carried out. In terms of the potential hematological toxicity, the mice’s blood was collected 24 hours before and after the fourth administration for the routine blood test and blood biochemical test after the last administration. It can be observed from **Supplementary Figure 4** and **Figure 5E** that there was no obvious hematologic toxicity in the two dose of YG-003D3. All the indicators (except AST) from the experimental group were not statistically different from the IgG control group. However, the AST content in the median-dose YG-003D3 group decreased rather than increased. This result may also be related to individual differences and the decrease was not significant, which did not indicate that the liver function of the mice was damaged. To further study the anti-tumor immune response induced by drugs. Blood and tumor tissues were collected at the end of the experiment. Subsequently, the ratio of CD45^+^cells, CD3^+^ T cells, CD8^+^ T cells, CD4^+^ T cells and Treg cells in the tumor and peripheral blood was analyzed. **Figure 5F** shows that YG-003D3 changed the ratio of T cell subtypes in the tumor microenvironment. According to the statistical results, compared with the IgG control group, YG-003D3 (6 mg/kg) group could induce more CD45^+^ cells, CD3^+^ T cells and CD8^+^ T cells to infiltrate into the tumor tissue (p=0.0402, 0.0205, 0.0202, respectively), and the proportion of CD4^+^ T and Treg cells with immunosuppressive function did not change significantly compared with the control group. Therefore, we hypothesized that the increase of CD3^+^ T cells (total T cells), especially CD8^+^ killer T cells, in the tumor microenvironment is one of the important molecular characteristics of the anti-tumor effect of YG-003D3 (6 mg/kg) group (the original results of immune cells infiltrated into tumor tissue in each mouse are shown in **Supplementary Materials 2**). Similar results were also found in peripheral blood (**Figure 5G**). Compared with IgG control group, YG-003D3 (6 mg/kg) group could induce more CD3^+^ T cells, CD8^+^ T cells and CD4^+^ T cells in peripheral blood (p=0.0080, 0.0028, 0.0165, respectively), while the proportion of Treg cells with immunosuppressive function did not change significantly compared with the control group. Not quite as expected is that the total number of CD45^+^ cells in the YG-003D3 (6 mg/kg) group was lower than that in the control group. We speculated that due to technical problems, the red blood cell lysis operation was not well completed when processing this group of blood samples (the original results of immune cells in peripheral blood of each mouse are shown in **Supplementary Materials 3**). These results demonstrate that YG-003D3 can not only induce an increase in the proportion of T cells that exert tumor killing effect in peripheral blood, but also effectively induce an increase in the proportion of total T lymphocyte infiltrating in tumor tissues.

Secondly, to further compare the anti-tumor differences between the YG-003D3 and the combination groups. In fact, we set YG-003D3(2mg/kg) and FV78+MIL-80(1mg/kg+1mg/kg) during the above experiment and regarded as group 1, in order to see the anti-tumor effect of the combination group with the same dose of double antibodies. However, a single dose does not tell the whole story. Therefore, we set up group 2 of YG-003D3(4mg/kg) and FV78+MIL-80(2mg/kg+2mg/kg) to compare whether the BsAbs group has the potential to be superior to the combination group at two different doses. As the results shown in **Figure 6A**, since the tumor growth of mice in group 2 was slow, we stopped administration after the fourth administration and recorded the tumor growth trend and mouse weight change. the result showed that that body weight of the mice increase slightly over time (**supplementary Figure 5**). The tumor growth trend is shown in **Figure 6B**. According to the statistical results, both in the group 1 and the group 2, the inhibitory effect of YG-003D3 on tumor was significantly better than that of the combined group (P<0.05, P<0.01, respectively). Moreover, In group 1, the tumor weight after treatment with 2mg/kgYG-003D3 and 1mg/kgFV78+1mg/kgMIL-80 was 0.9576 ± 0.21g and 1.77 ± 0.4645g respectively. In group 2, the tumor weight after treatment with 4mg/kg YG-003D3 and 2mg/kg FV78+2mg/kg MIL-80 was 0.5143 + 0.2005g and 1.458 ± 0.4972g respectively. Although there was no statistically significant difference between the two groups in terms of the anti-tumor effect of the BsAbs group and that of the combination group at the same dose, we could clearly see that the anti-tumor effect of the BsAbs group was more pronounced, this is consistent with the results of the tumor volume inhibition rates of the drugs described above. Of note, In group 2, among the mice treated with 4mg/kg YG-003D3, one mouse tumor completely subsided before the fourth administration, but no tumor regression was found in the combined administration group. In conclusion, the above results indicate that YG-003D3 not only exerts anti-tumor effect by activating tumor and peripheral blood immune cells, but also has superior anti-tumor effect than the combination group under the same dose.

## Discussion

Bispecific antibodies are regarded as the next generation of antibody drugs (31, 32), they are a class of genetically engineered antibodies that do not exist naturally, it is produced by cell fusion or recombinant DNA technology (33, 34). Because of its specificity and bi-function, it has become a research hotspot in the field of antibody engineering and has a broad application prospect in the fields of tumor therapy and autoimmune diseases (34–36). Compared with the monoclonal antibody, the bispecific antibody has an additional specific antigen-binding site (37), which makes them more specific, more accurate in targeting tumor cells, and less toxic to off-target cells, there are four major advantages of BsAb including (1) BsAb has two antigen-binding sites, which allows them to bridge the tumor cells and immune cells, reorient immune cells, and recruit immune cells to the periphery of tumor cells and enhance the anti-tumor effect (37–39); (2) BsAb can block/activate two different immune signal pathways downstream at the same time to enhance the cytotoxicity of tumor cells (39); (3) BsAb can bind to two different cell surface antigens and may potentially increase binding specificity and reduce side effects such as off-target (40–43). (4) BsAb offers several benefits in terms of manufacture, preparation, and drug declaration, and it is less expensive than antibody combinations (44, 45). Therefore, bispecific antibodies will undoubtedly be popular in the future. So far, there are three bispecific antibody medications on the market: Blinatumomab, Amivantamab, and Tebentafusp (46). Although they benefit from the above-mentioned benefits, the structural diversity of BsAb and the corresponding pharmaceutical properties such as pharmacokinetics and immunogenicity have certain uncertainties compared with conventional antibodies. In this study, we constructed PD-1/LAG-3 bispecific antibodies with a ‘knob-into-hole’ structure, which promotes the formation of heavy chain heterodimers only by mutating Fc and the related structure and pharmacological properties are more similar to monoclonal antibodies.

In recent years, antibody drugs targeting PD-1/PD-L1 have made great breakthroughs in clinical practice, and are known as the most successful ‘broad-spectrum anti-cancer drug target in history (29, 47, 48). However, PD-1 antibody monotherapy is limited (49, 50). It is well known that T-cell activity is sophisticatedly regulated by multiple costimulatory/inhibitory signals (51–54). Thus, the combined targeting of different signaling pathways is being actively explored in ongoing clinical trials, in which PD-1/LAG-3 antibody combination therapy has shown more promising clinical benefits than single therapy (14, 15). Here, we describe a novel PD-1/LAG-3 bispecific antibody (YG-003D3) with a desirable co-targeting strategy. Based on design rationales, this molecule is speculated to elicit its distinctive antitumor profile through at least three mechanisms of action: (1) anti-PD-1 blocks the PD-1/PD-L1 interaction, alleviating T-cell exhaustion; (2) Targeting PD-1/LAG-3 on a T cell surface, blocking LAG-3/MHC-II binding and reversed the drug resistance of anti-PD-1 monotherapy to some extent; (3) YG-003D3 can enrich T cells and alleviate the inhibition of immune detection points by bridging PD-1^+^ cells and LAG-3^+^ cells. These mechanisms collectively explain the superior anti-tumor efficacy of the PD-1/LAG-3 bispecific antibody compared with anti-PD-1 and anti-LAG-3 monotherapy or their combination in our *in vitro* functional studies and *in vivo* models. Compared with the currently studied PD-1(PD-L1) and LAG-3 bispecific antibodies (27), some researchers have used anti-LAG-3 antibody in the C-terminal connected to an anti-PD-L1 nanobody to form a double antibody structure, this structure theoretically has larger molecular weight, and the potential immunogenicity, the expression level of industrial large-scale production and the stability of the protein all need to be considered in the follow-up study. In addition, some researchers have designed BsAb by mutating the antibody CH3 to form the binding domain of LAG-3 antigen, but this has largely changed the structure of the antibody, especially the CH3. Similarly, the immunogenicity and the specificity are also need to be considered (55). In this study, we used the ‘ Knob-into-Hole ‘, which is closest to the natural antibody structure, to design the PD-1/LAG-3 BsAb. In theory, this structure has excellent druggability, and one of Roche ‘s BsAb products Hemlibra is also based on the concept of ‘ Knob-into-Hole ‘ structure (56).This is enough to prove that the bispecific antibody of ‘ Knob-into-Hole ‘ has unique advantages. Furthermore, considering that the two targets in this study are both molecules on the surface of immune cells, we mutated Fc(N297A) by genetic engineering to eliminate Fc-mediated ADCC effects and reduce the potential cytotoxicity of drugs.

The tumor microenvironment consists of not only tumor cells, but also immune cells, tumor-related stromal cells, etc. in and around the tumor (57, 58). The strategy to make more active immune cells gather in the tumor microenvironment is an important method to cure tumors (59–61). In this study, we designed a novel PD-1/LAG-3 bispecific antibody YG-003D3 and verified that BsAb could bridge PD-1^+^ and LAG-3^+^ cells simultaneously. Our results in **Figure 4A** support the ability of YG-003D3 to bridge cells expressing PD-1^+^ and LAG-3^+^, respectively. Moreover, YG-003D3 is also significantly better than the monoclonal antibody group in PBMCs binding (**Figure 2G**), which may be due to the simultaneous expression of these two target antigens on immune cells, while only the unique structure of BsAbs can ensure that two antigen binding sites can simultaneously recognize double antigen molecules on the same cell. Therefore, we also did PBMCs activation experiment, mixed lymphocyte experiment and tumor cell killing experiment *in vitro*. All experiments showed that YG-003D3 could activate immune cells to release cytokines and kill tumor cells more effectively. Therefore, we speculate that YG-003D3 may exert a more effective blocking effect by bridging the double-antigen molecules on different immune cells or simultaneously binding the double-antigen molecules on the same cell. In the PBMCs activation assay, YG-003D3 induced stronger IFN-γ, IL-6, and TNF-α secretion compared to that induced by its parental anti-PD-1 or anti-LAG-3 antibodies. Although IL-6 is not the major cytokine secreted by T cells, some literature reports that IL-6 may be secreted by monocytes, macrophages, and dendritic cells (62–64). Therefore, it can be speculated that YG-003D3 may affect other immune cells, either directly or indirectly, and then induce the expression of IL-6. We still have no clear evidence at present, and we hope to consider it in the future studies. We evaluated the *in vivo* activity of YG-003D3 in the PD-1/LAG-3 double knock-in mouse model bearing MC38(hPD-L1). YG-003D3 significantly inhibited MC38(hPD-L1) tumor growth and led to the increased number of T cells that exert tumor killing effect in the tumors and blood. This is an important sign of the anti-tumor effect of bispecific antibody against PD-1/LAG-3.

Taken together, YG-003D3 is a PD-1/LAG-3 BsAb that targets and inhibits both PD-1 and LAG-3 pathways and has the potential to overcome primary and acquired anti-PD-(L)1 resistance *via* a synergistic effect. YG-003D3 has enhanced anti-tumor efficacy compared with monotherapy without obvious hematologic toxicity. Our team is also developing drugs based on YG-003D3, which will undoubtedly be further proven clinically as a potential anti-tumor drug with the hope of bringing clinical benefits to more cancer patients.

## Data availability statement

The original contributions presented in the study are included in the article/**supplementary materials**. Further inquiries can be directed to the corresponding authors.

## Ethics statement

The study protocol was approved by animal laboratory of Laboratory Animal Center Academy of Military Medical Sciences.

## Author contributions

LL, XG, and CR conceived and designed this study. The anti-tumor effect of YG-003D3 was verified by NS and YZ. XJ supplements the missing experimental data. YL and YZ critically reviewed and revised the manuscript. The contributions of NS and YZ to this manuscript are equal. All authors contributed to the article and approved the submitted version.

## Funding

We acknowledge the National Natural Science Foundation of China (Grant No.82204262, 21976210, 81773179, 82071399, 81472355), Key Research and Development Program of Hunan (Grant no. 2022SK2055), Provincial Natural Science Foundation of Hunan(Grant no. 2022JJ30931, 2020JJ4771), Incubation Project of Medical Science and Technology Youth Training Program (Item Number: 20QNPY143) and Beijing Nova Program (Grant Number Z211100002121020) for providing financial support for this research.

## Conflict of interest

The authors declare that the research was conducted in the absence of any commercial or financial relationships that could be construed as a potential conflict of interest.

## Publisher’s note

All claims expressed in this article are solely those of the authors and do not necessarily represent those of their affiliated organizations, or those of the publisher, the editors and the reviewers. Any product that may be evaluated in this article, or claim that may be made by its manufacturer, is not guaranteed or endorsed by the publisher.

## Abbreviations

Treg, Regulatory T cells; Fv, Fragment of the variable region; EC50, Concentration for 50% of maximal effect; ELISA, Enzyme-Linked Immunosorbent Assay; HPLC, High-Performance Liquid Chromatography; SEC, Size Exclusion Chromatography; HIC, Hydrophobic interaction chromatography; MFI, Median Fluorescence Intensity; GSH, Glutathione; BLI, Biolayer interferometry; PBMCs, Peripheral Blood Mononuclear Cells; TGI, Tumor growth inhibition; TME, Tumor Microenvironment; SPF, Specific Pathogen Free; FBS, Fetal Bovine Serum; SDS-PAGE, Sodium Dodecyl Sulfate Polyacrylamide Gel Electrophoresis; ADCC, antibody-dependent cell-mediated cytotoxicity.
