# Supplementary material for: PD-1/LAG-3 bispecific antibody potentiates T cell activation and increases antitumor efficacy
Source: Front Immunol. 2022 Nov 28;13:1047610. doi: 10.3389/fimmu.2022.1047610 (PMC9742559; doi:10.3389/fimmu.2022.1047610)
Supplement: Supplementary file 1 [file DataSheet_1.docx]

Supplementary Material

# Supplementary Figures


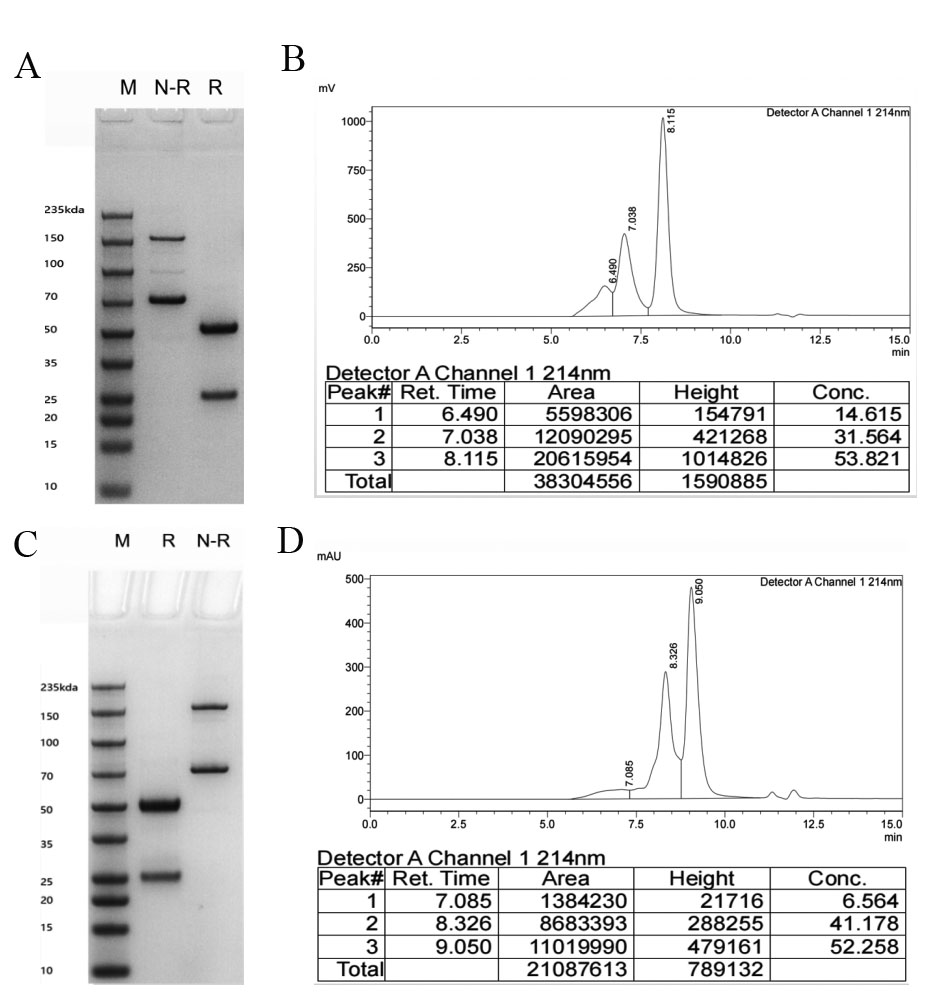


**Supplementary Figure 1.** The purified semi-antibody components were analyzed by SDS-PAGE and SEC**.** (A) The purified Knob protein was examined by SDS-PAGE. (B) The purified Knob protein was examined by SEC. (C) The purified Hole protein was examined by SDS-PAGE. (D) The purified Hole protein was examined by SEC. Since Knob/Hole half-antibodies are prone to self-aggregation, two bands will appear on SDS-PAGE in the non-reducing state. There may be multiple peaks in the SEC chromatogram.


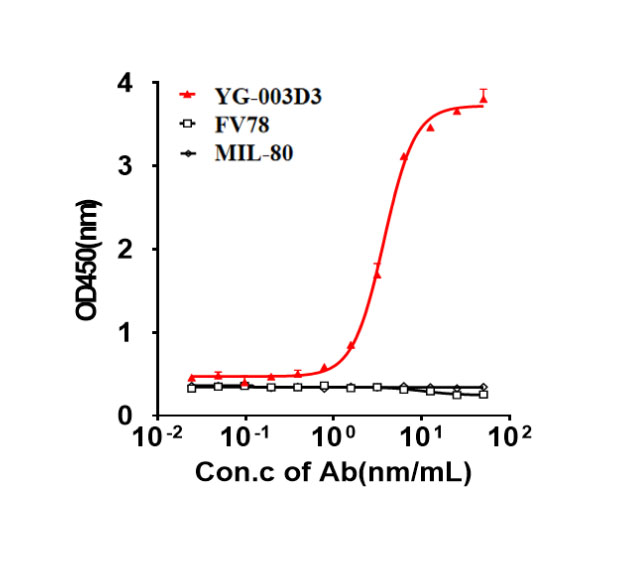


**Supplementary Figure 2.** Double-antigen sandwich ELISA was used to verify that the bispecific antibody could simultaneously target two targets of LAG-3/PD-1.


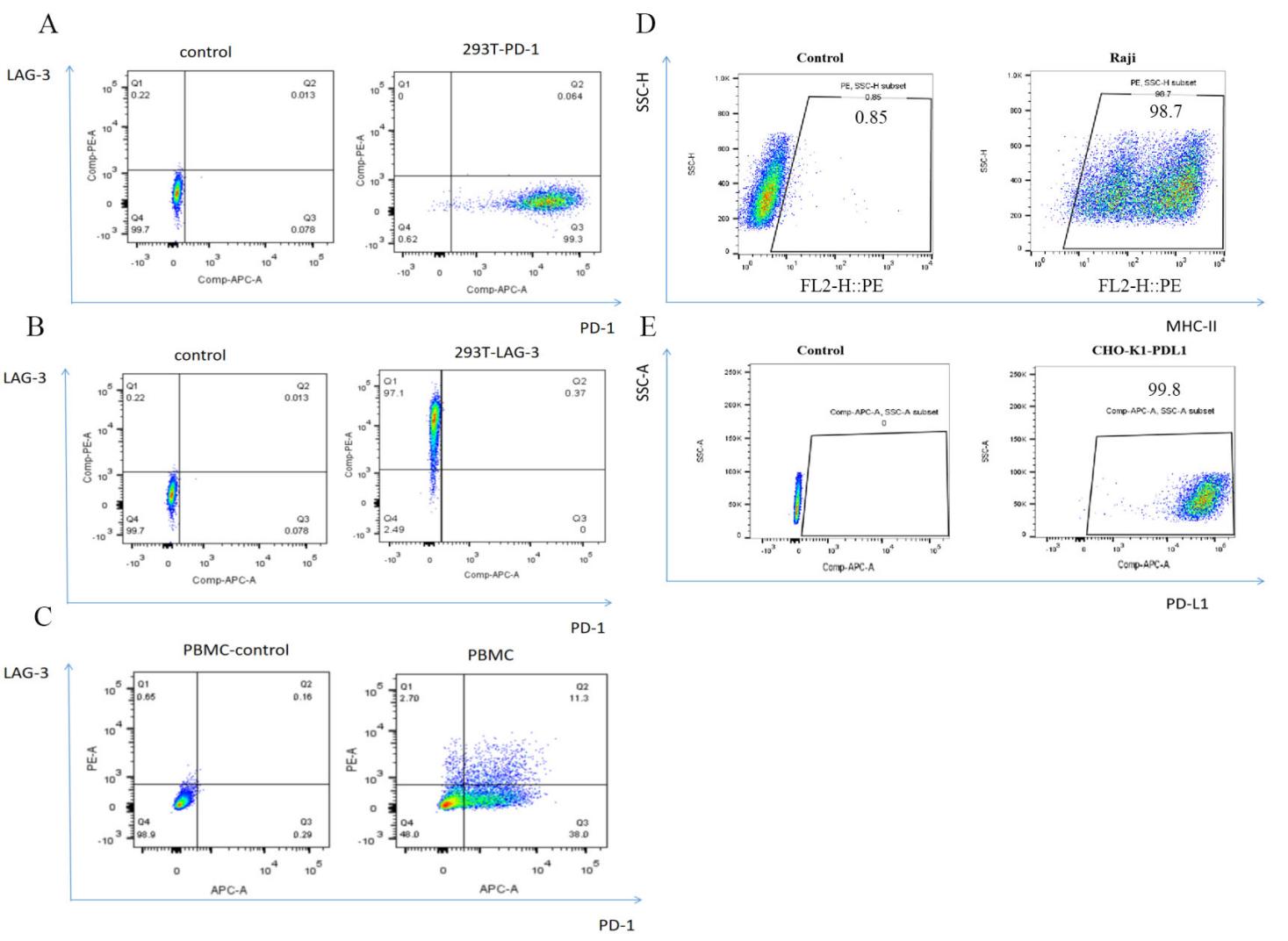


**Supplementary Figure 3.** Cell line construction and identification. A. Construction of 293T cell line expressing LAG-3. B. Construction of 293T cell line expressing PD-1. C. Simultaneous expression of LAG-3/PD-1 on PBMC. D. Raji cells expressing MHC-II. E. CHO-K1(hPD-L1) cells expressing human PD-L1.


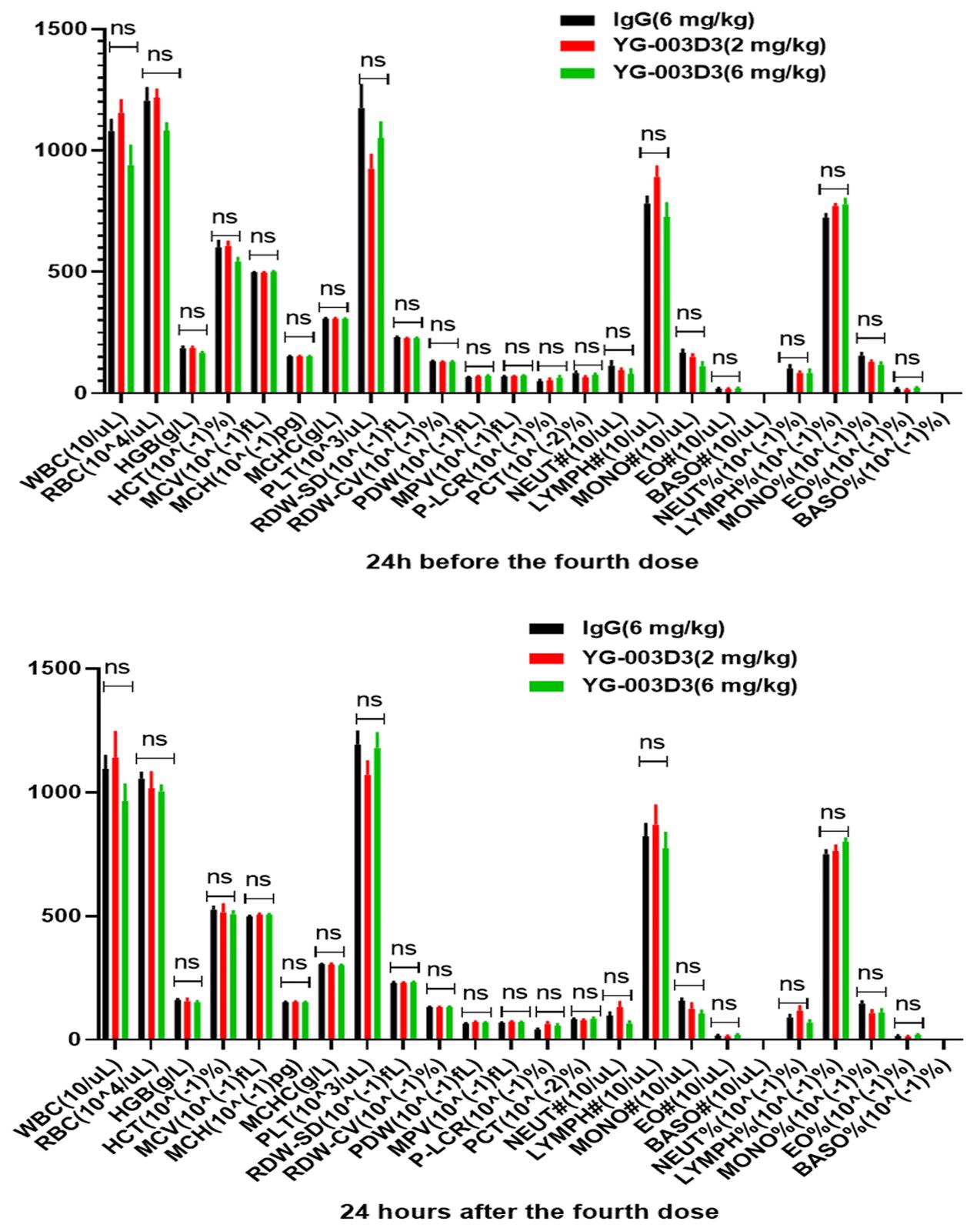


**Supplementary Figure 4.**  Blood routine examination 24 hours before and after the fourth administration.

**Supplementary Figure 5.** Mouse body weight changes during antibody drug therapy.
