# Supplementary material for: PD-1/LAG-3 bispecific antibody potentiates T cell activation and increases antitumor efficacy
Source: Front Immunol. 2022 Nov 28;13:1047610. doi: 10.3389/fimmu.2022.1047610 (PMC9742559; doi:10.3389/fimmu.2022.1047610)
Supplement: Supplementary file 2 [file DataSheet_2.pdf]

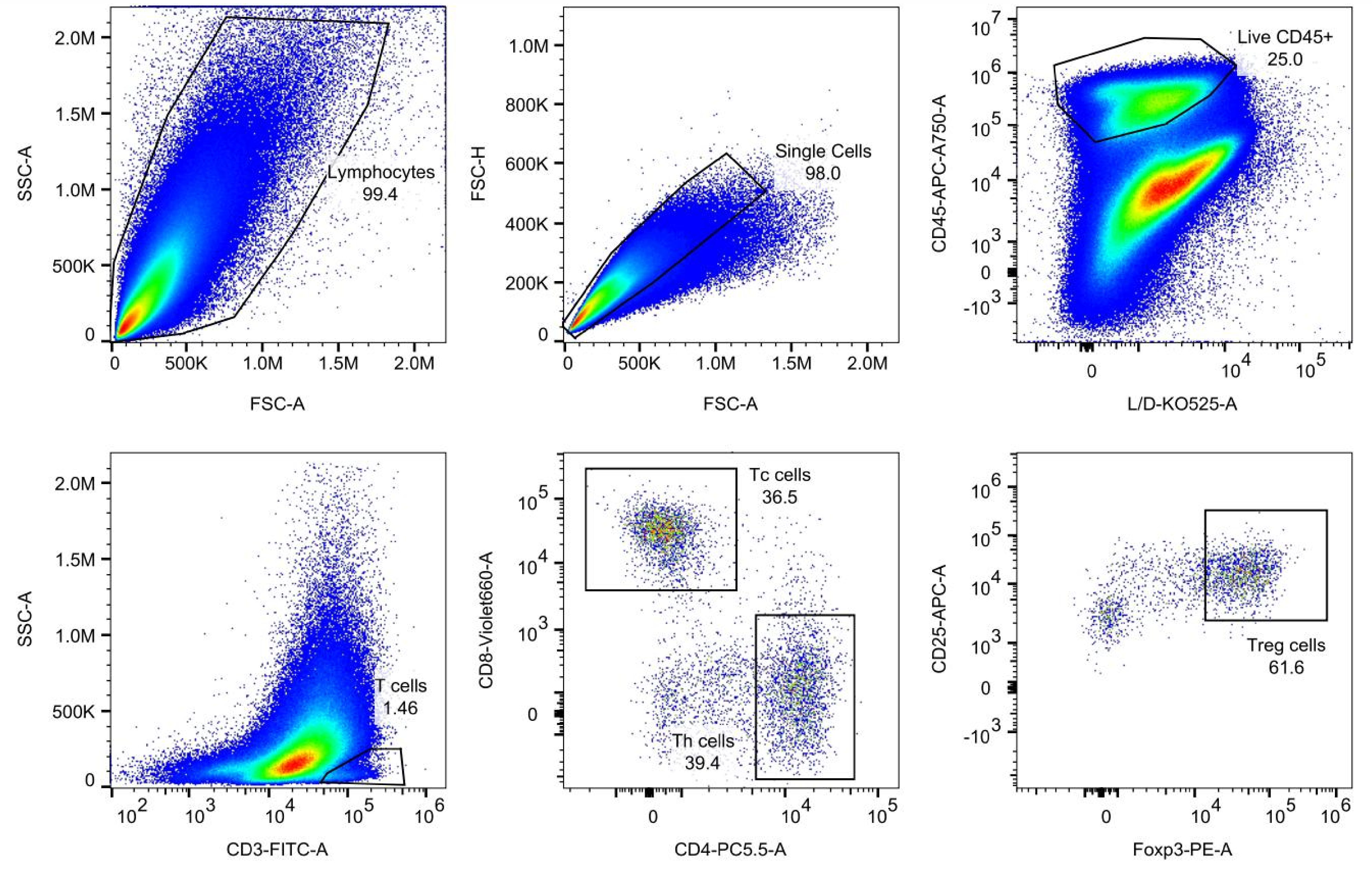

SSC-A
FSC-A
Lymphocytes
99.4
FSC-H
FSC-A
Single Cells
98.0
CD45-APC-A750-A
L/D-KO525-A
Live CD45+
25.0
SSC-A
CD3-FITC-A
T cells
1.46
CD8-Violet660-A
CD4-PC5.5-A
Tc cells
36.5
Th cells
39.4
CD25-APC-A
Foxp3-PE-A
Treg cells
61.6

IgG（6mg/kg）- 1

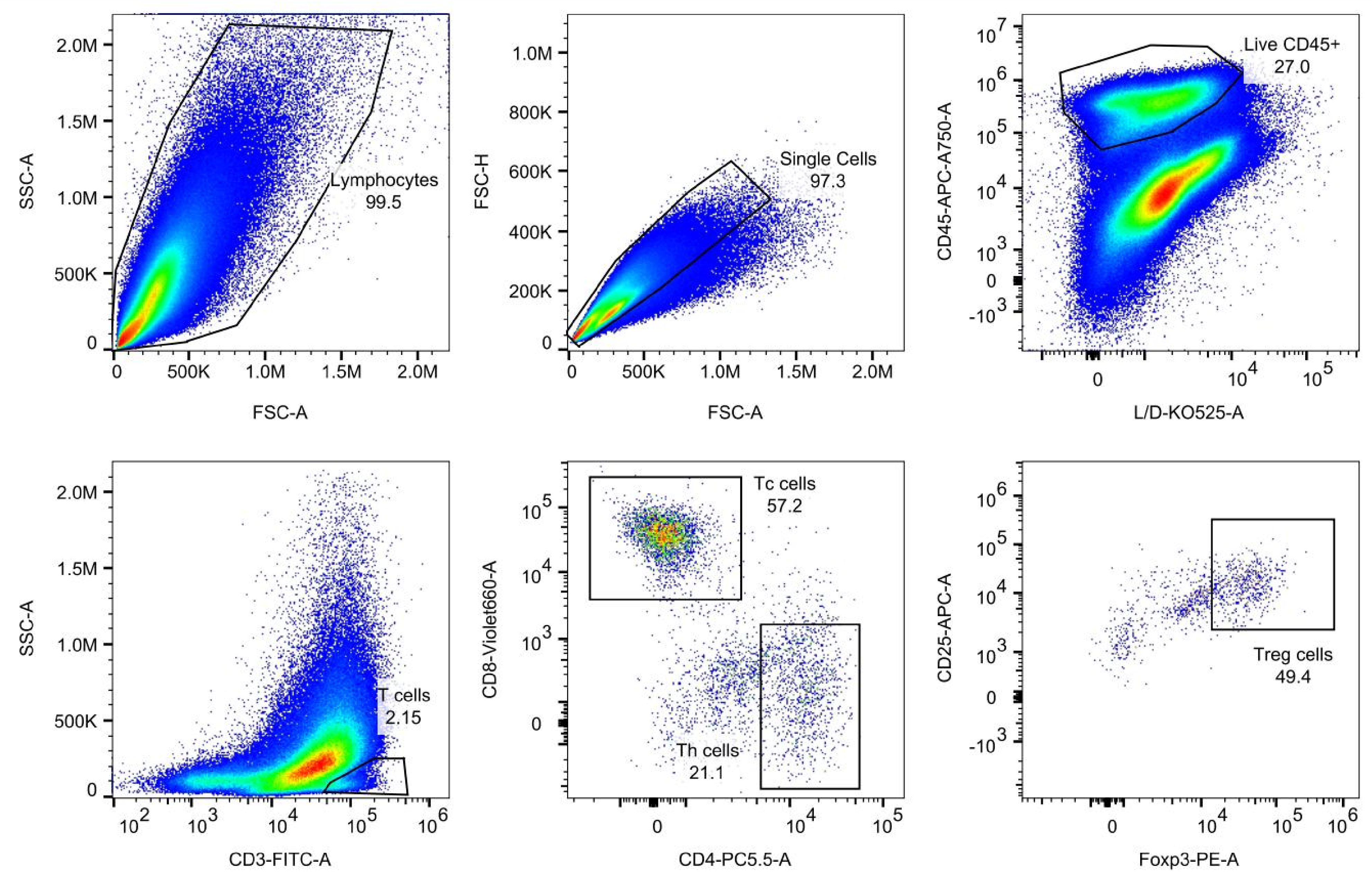

SSC-A
FSC-A
Lymphocytes
99.5
FSC-H
FSC-A
Single Cells
97.3
CD45-APC-A750-A
L/D-KO525-A
Live CD45+
27.0
SSC-A
CD3-FITC-A
T cells
2.15
CD8-Violet660-A
CD4-PC5.5-A
Tc cells
57.2
Th cells
21.1
CD25-APC-A
Foxp3-PE-A
Treg cells
49.4

IgG（6mg/kg）- 2

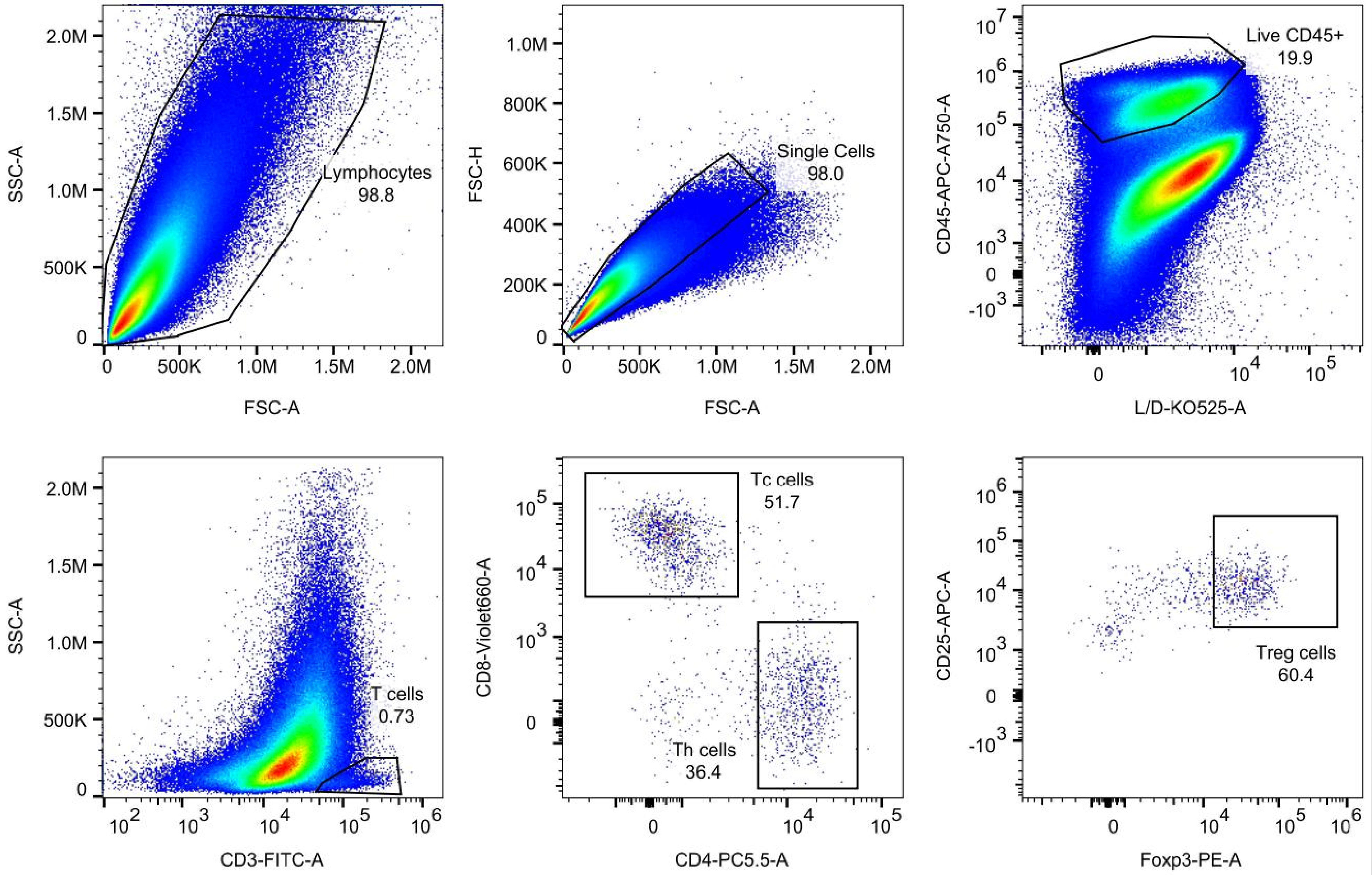
Lymphocytes
98.8
SSC-A
FSC-A
Single Cells
98.0
FSC-H
FSC-A
Live CD45+
19.9
CD45-APC-A750-A
L/D-KO525-A
T cells
0.73
SSC-A
CD3-FITC-A
Tc cells
51.7
Th cells
36.4
CD8-Violet660-A
CD4-PC5.5-A
Treg cells
60.4
CD25-APC-A
Foxp3-PE-A

IgG (6mg/kg) - 3

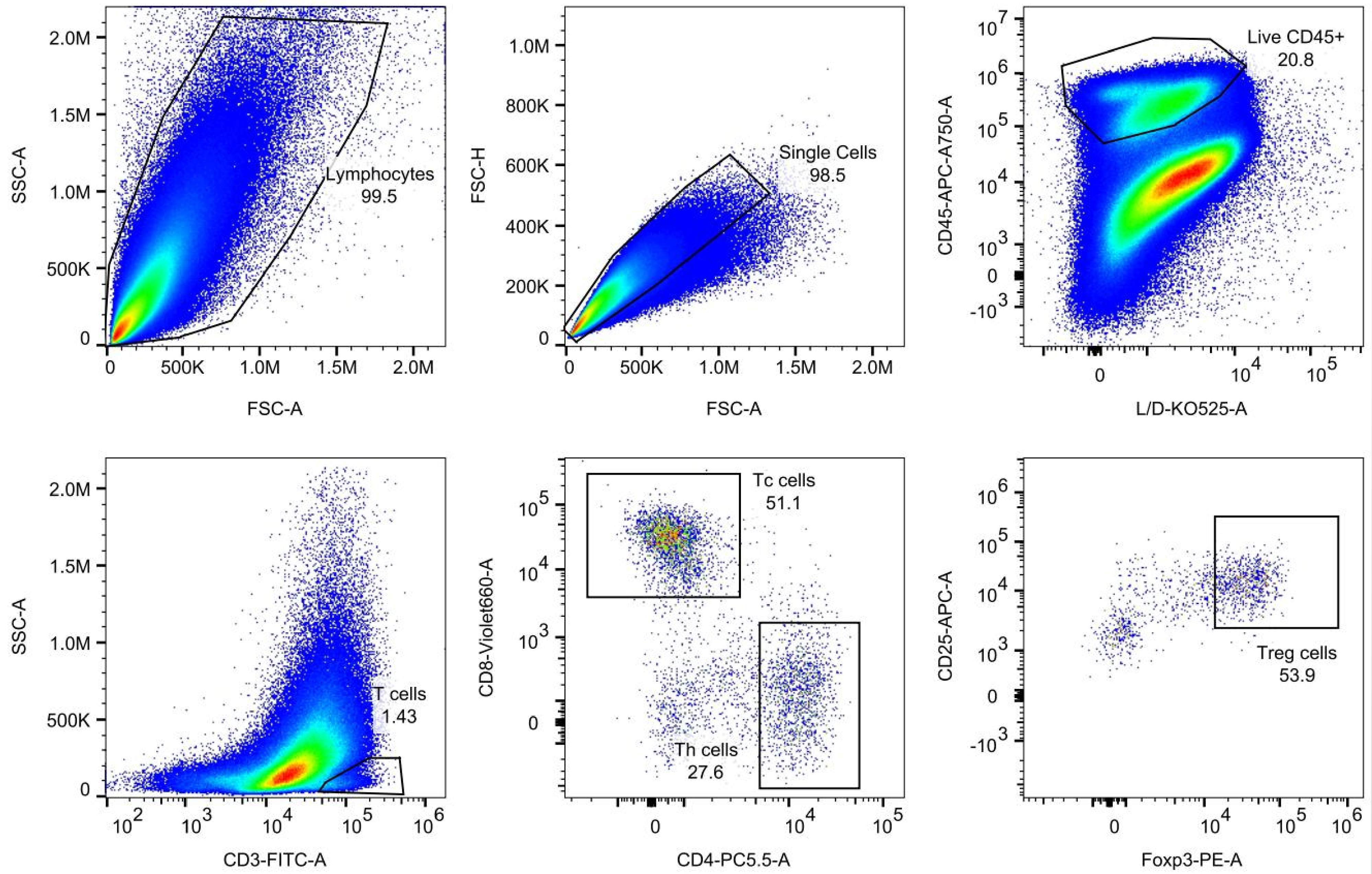
Lymphocytes
99.5
SSC-A
FSC-A
Single Cells
98.5
FSC-H
FSC-A
Live CD45+
20.8
CD45-APC-A750-A
L/D-KO525-A
T cells
1.43
SSC-A
CD3-FITC-A
Tc cells
51.1
Th cells
27.6
CD8-Violet660-A
CD4-PC5.5-A
Treg cells
53.9
CD25-APC-A
Foxp3-PE-A

IgG（6mg/kg）- 4

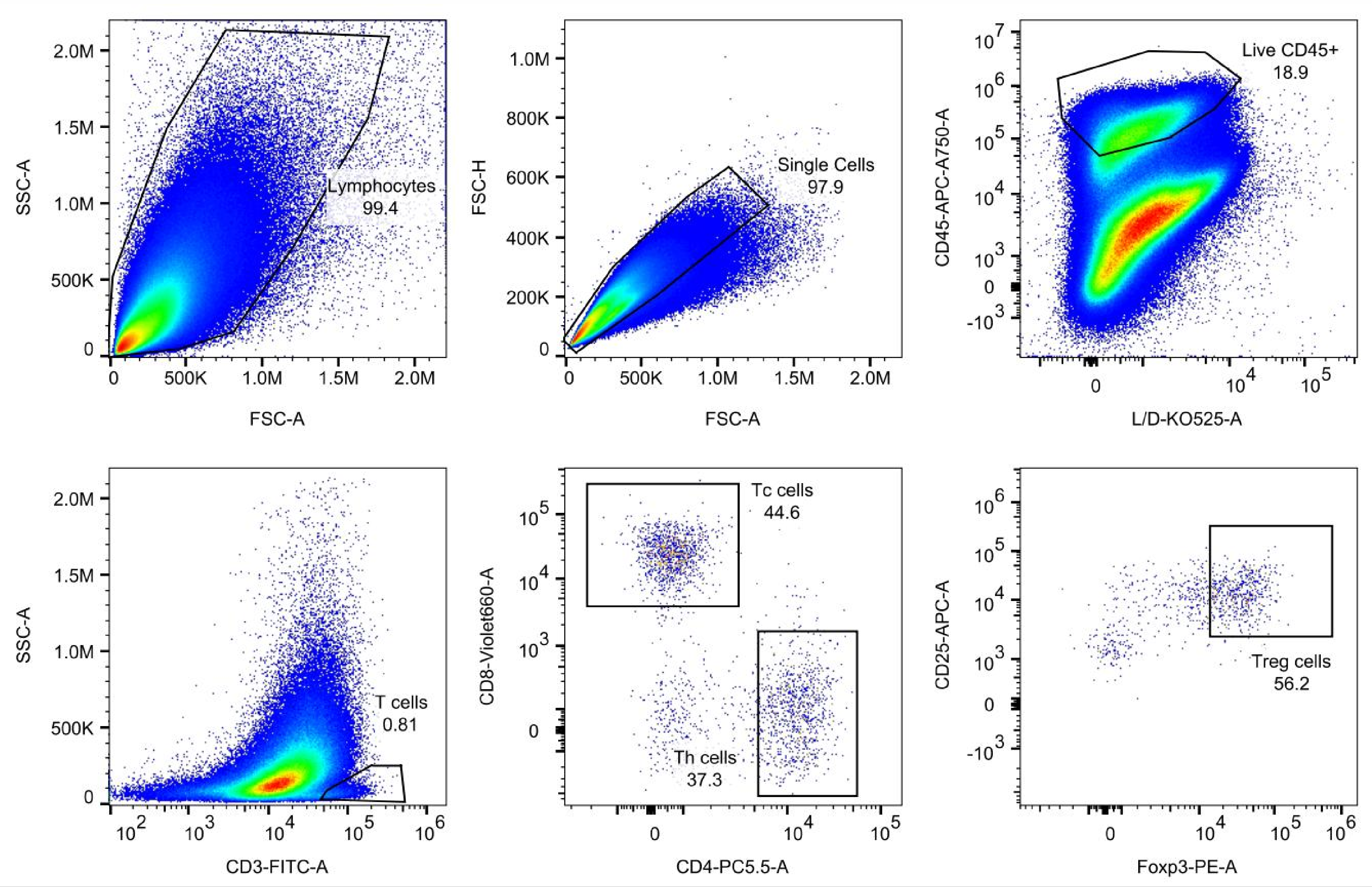

Lymphocytes
99.4
SSC-A
FSC-A
Single Cells
97.9
FSC-H
FSC-A
Live CD45+
18.9
CD45-APC-A750-A
L/D-KO525-A
T cells
0.81
SSC-A
CD3-FITC-A
Tc cells
44.6
Th cells
37.3
CD8-Violet660-A
CD4-PC5.5-A
Treg cells
56.2
CD25-APC-A
Foxp3-PE-A

IgG（6mg/kg）- 5

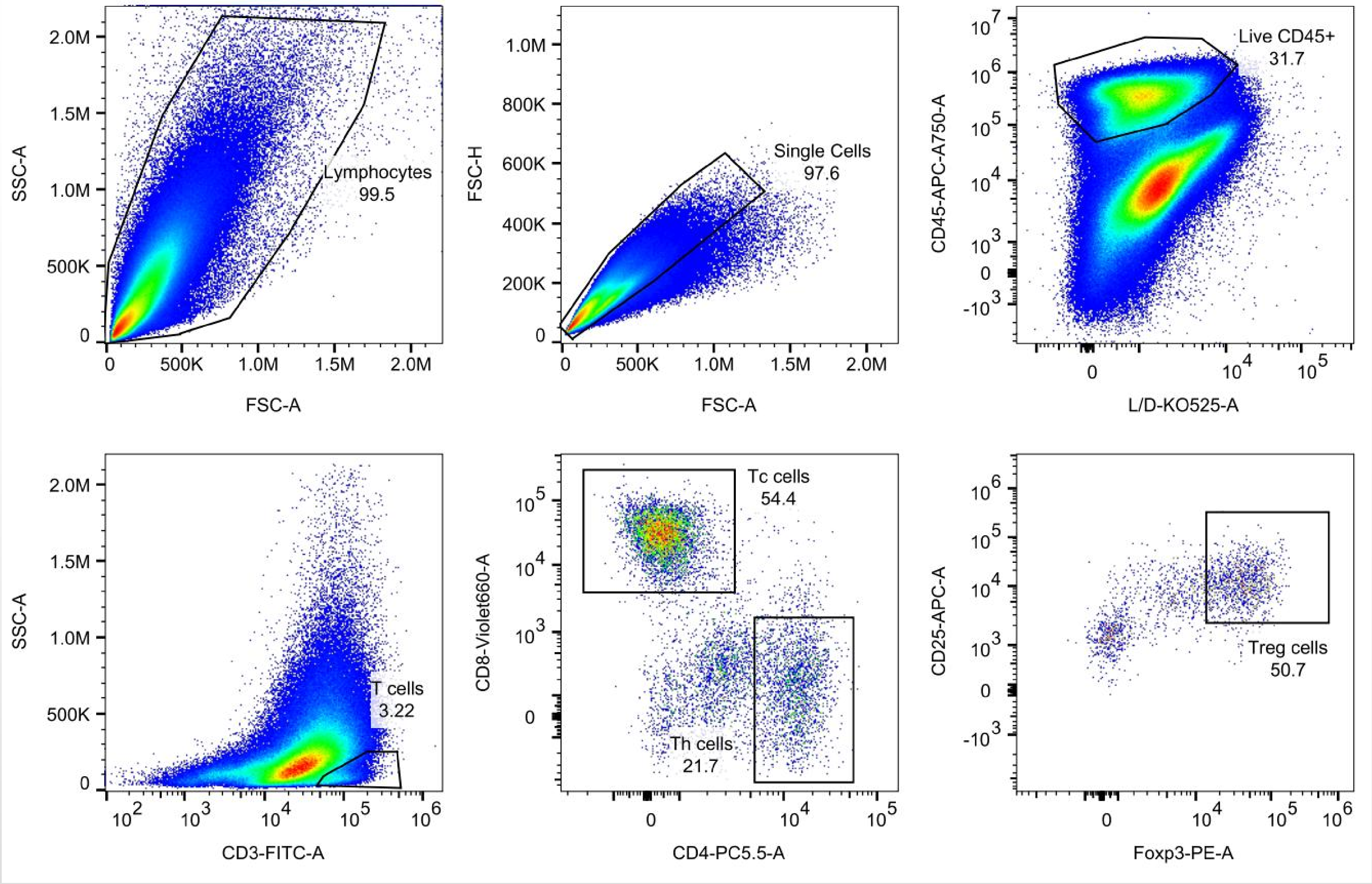

Lymphocytes
99.5
SSC-A
FSC-A
Single Cells
97.6
FSC-H
FSC-A
Live CD45+
31.7
CD45-APC-A750-A
L/D-KO525-A
T cells
3.22
SSC-A
CD3-FITC-A
Tc cells
54.4
Th cells
21.7
CD8-Violet660-A
CD4-PC5.5-A
Treg cells
50.7
CD25-APC-A
Foxp3-PE-A

IgG（6mg/kg）- 6

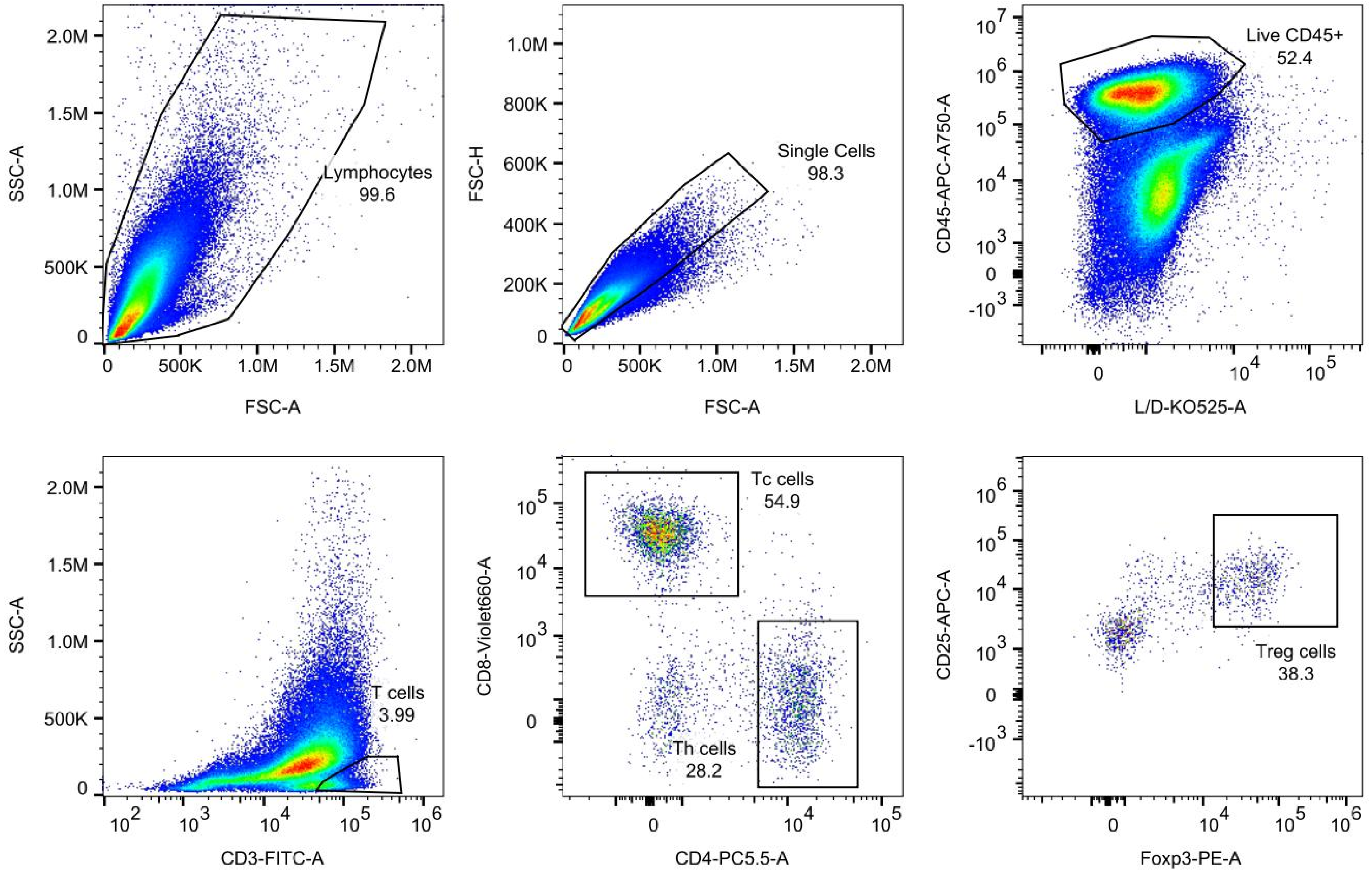

YG-003D3（2mg/kg）- 1

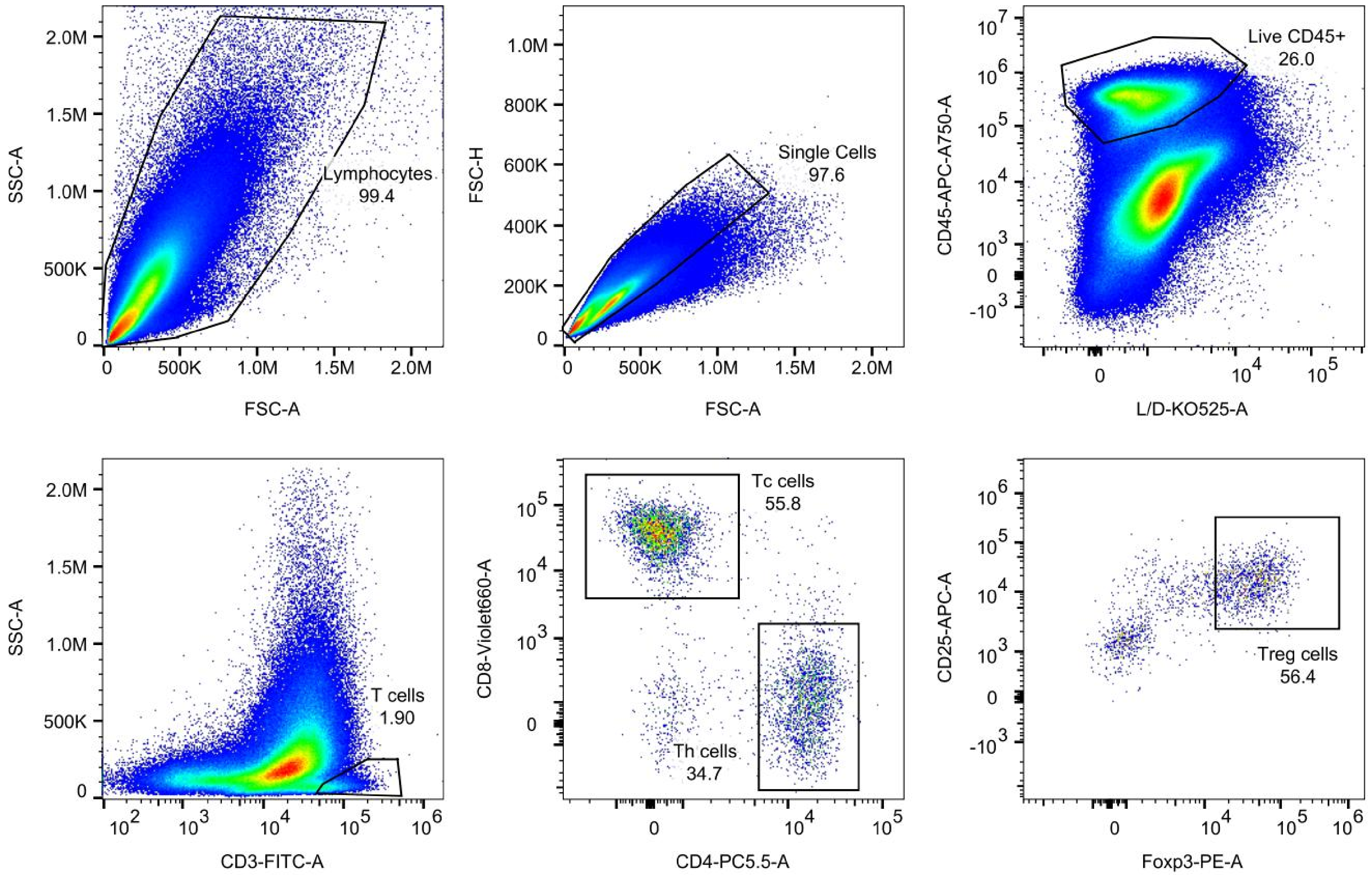

YG-003D3（2mg/kg）- 2

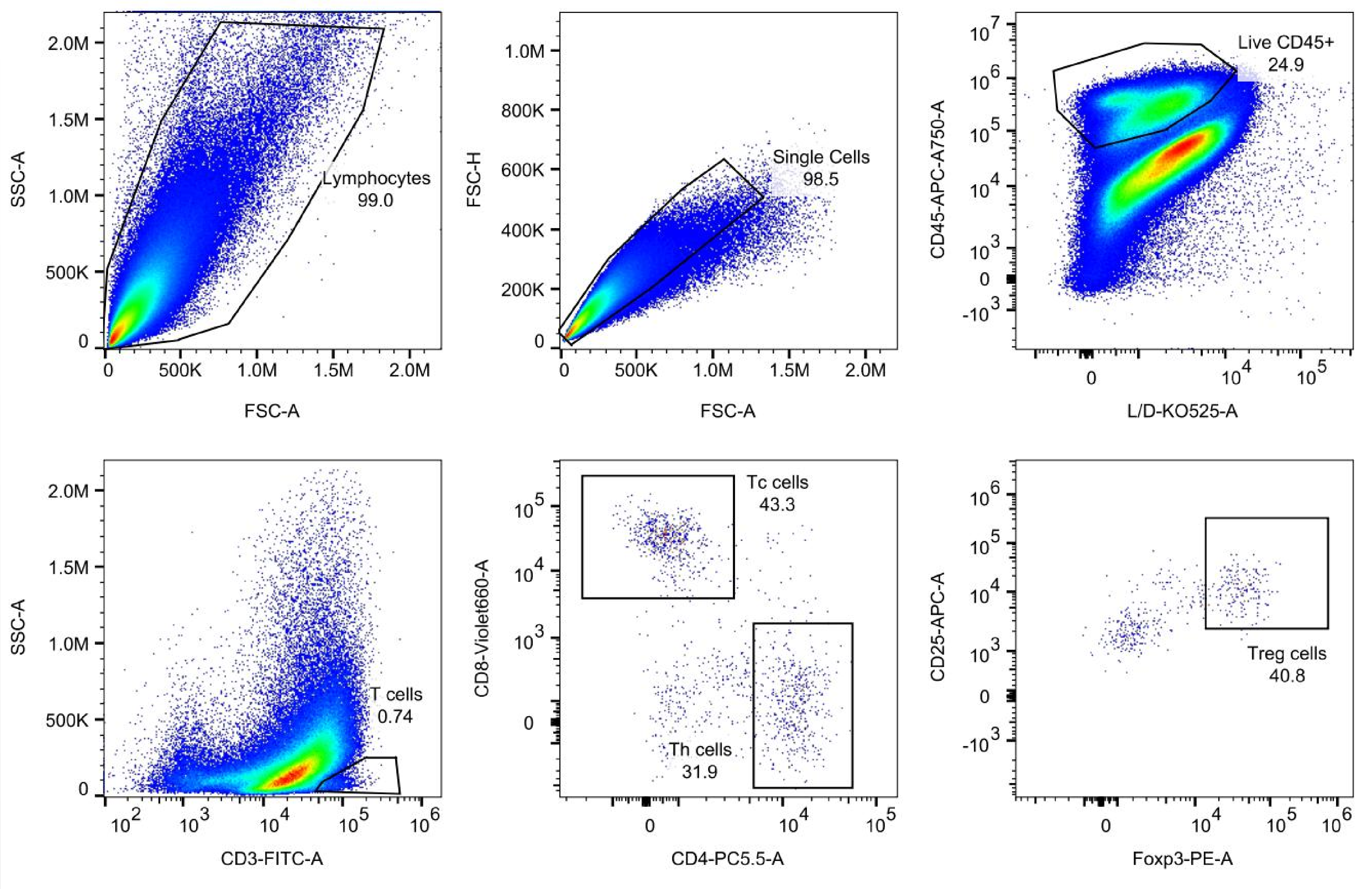

YG-003D3（2mg/kg）- 3

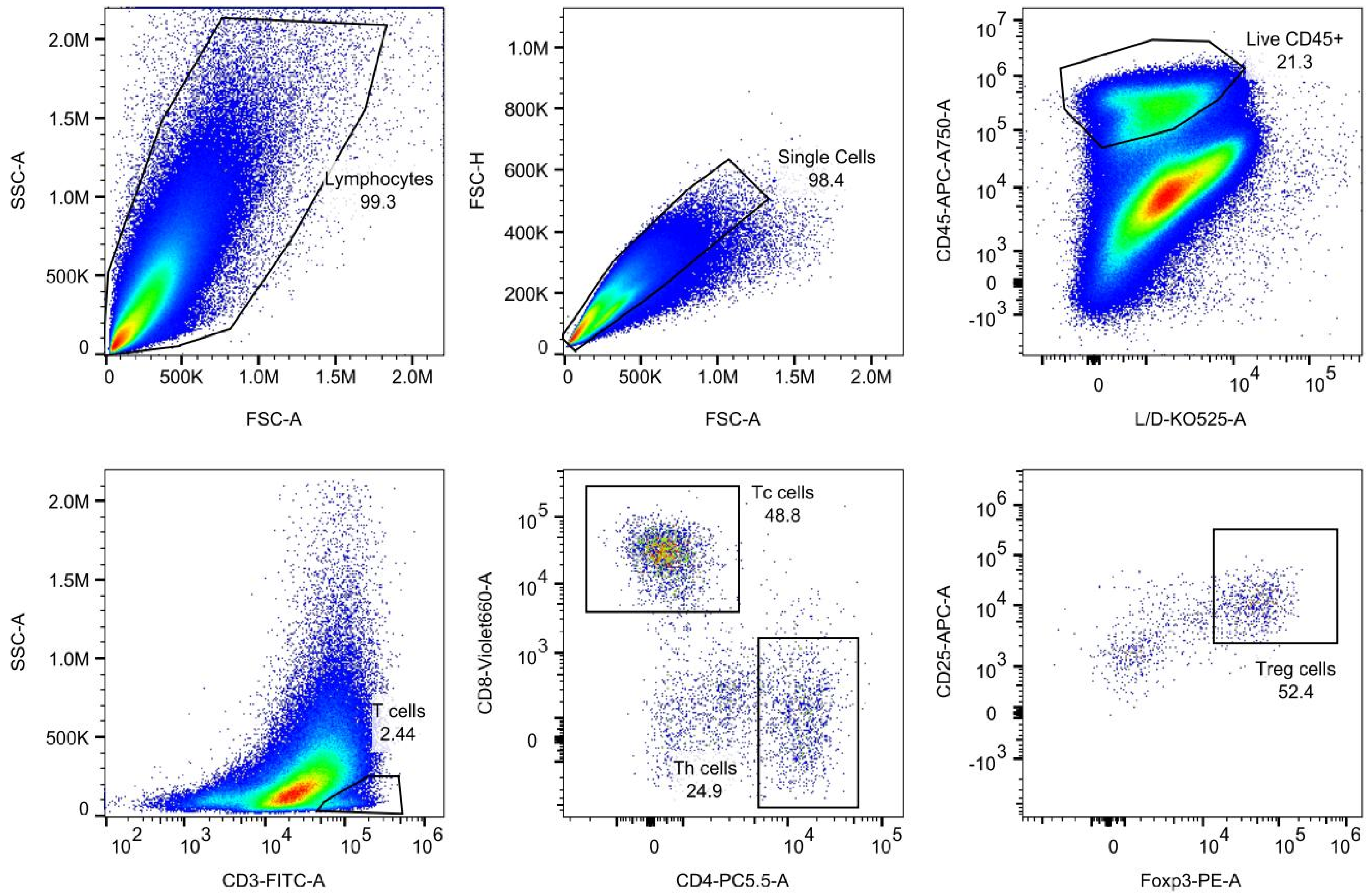

YG-003D3（2mg/kg）- 4

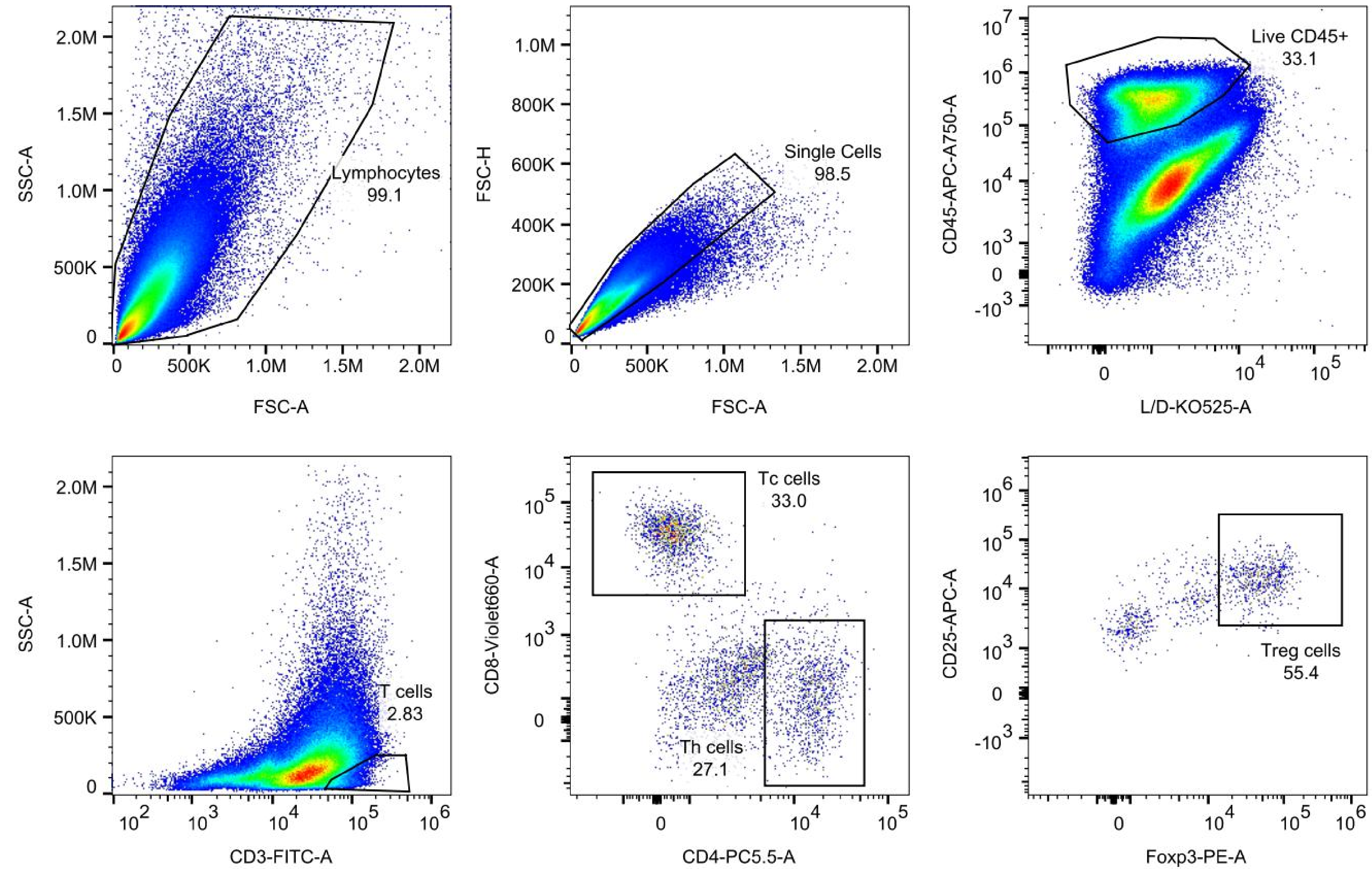

YG-003D3（2mg/kg）- 5

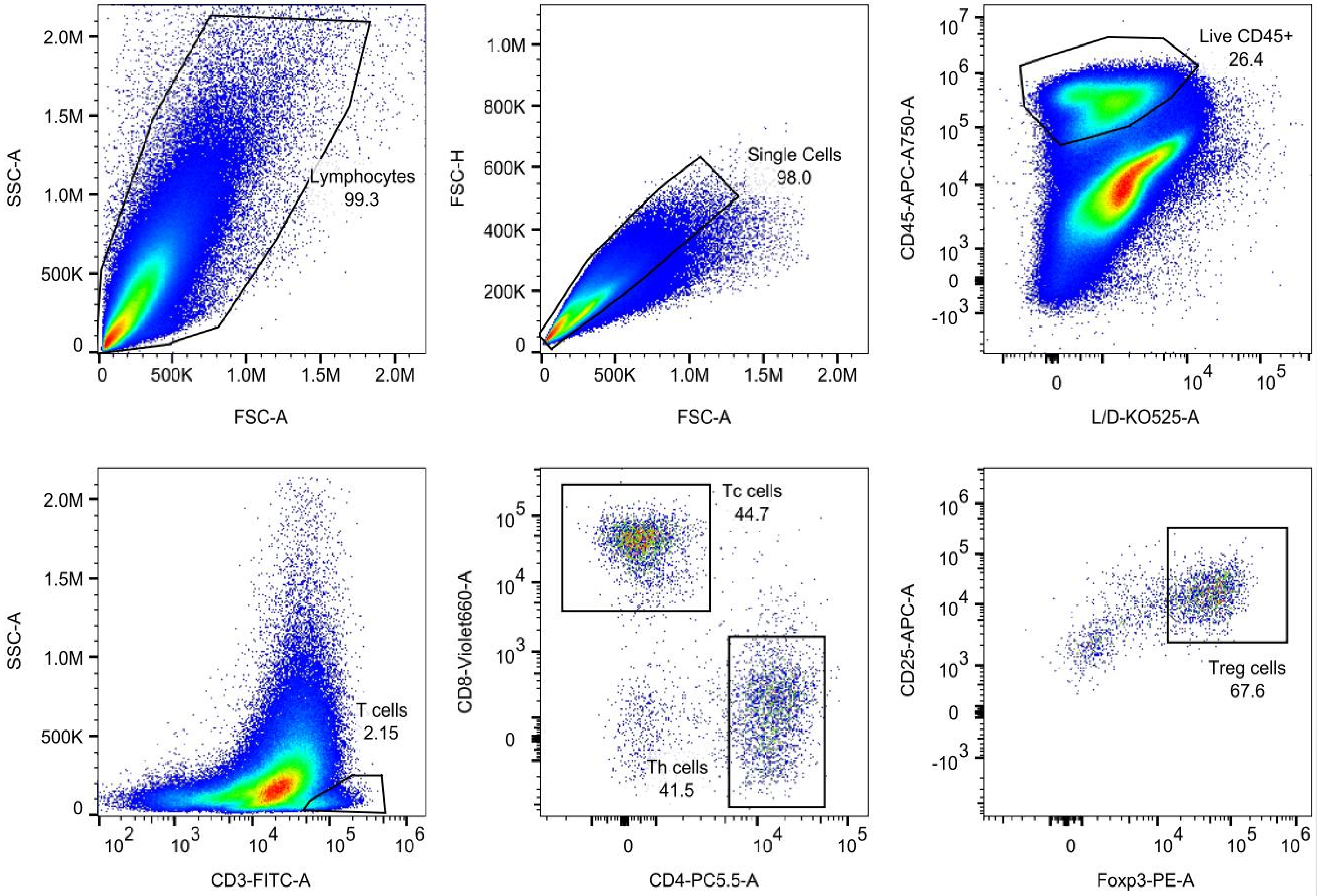

YG-003D3（2mg/kg）- 6

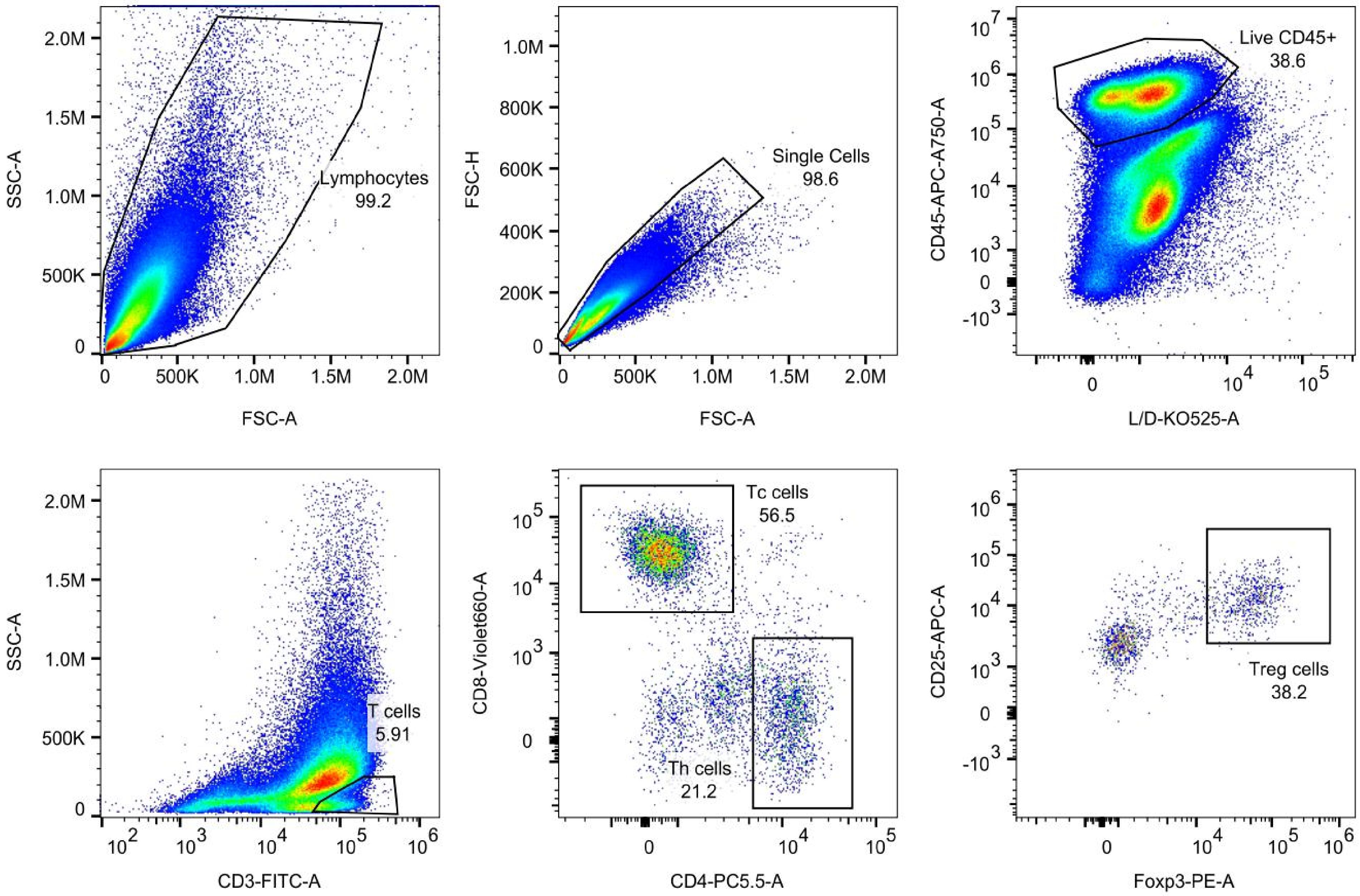

YG-003D3（6mg/kg）- 1

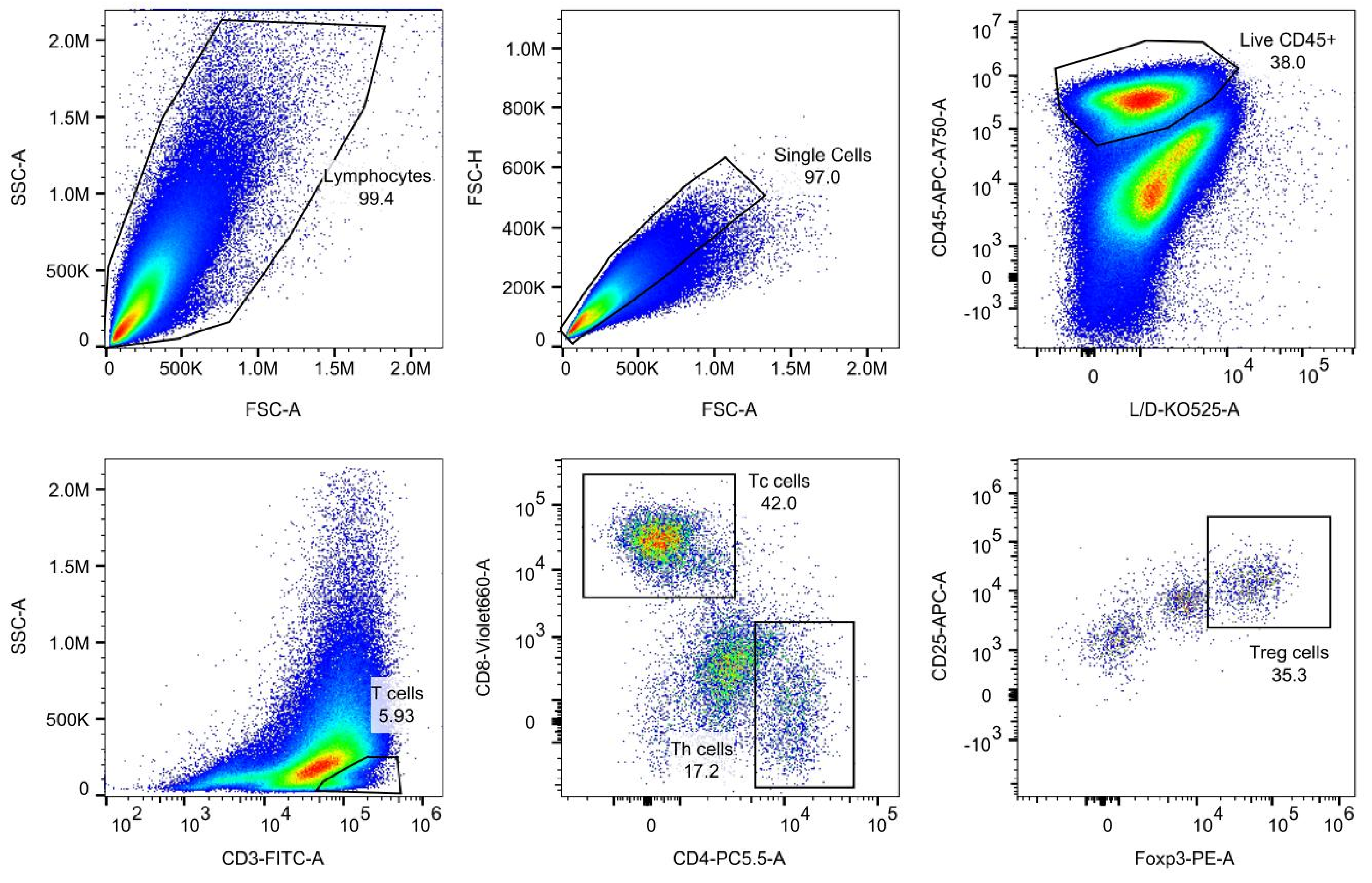

YG-003D3（6mg/kg）- 2

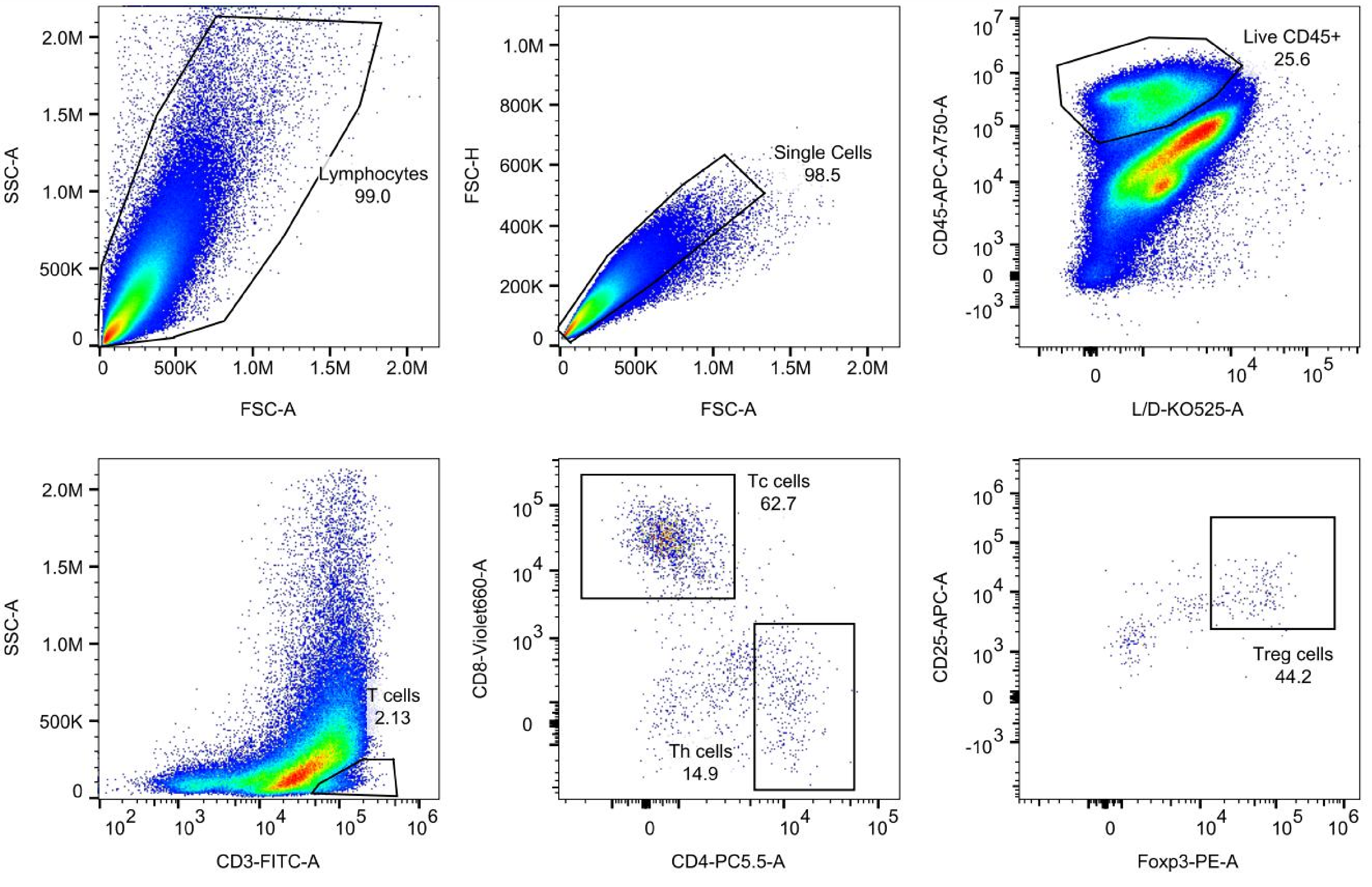

YG-003D3（6mg/kg）- 3
