# Supplementary material for: PD-1/LAG-3 bispecific antibody potentiates T cell activation and increases antitumor efficacy
Source: Front Immunol. 2022 Nov 28;13:1047610. doi: 10.3389/fimmu.2022.1047610 (PMC9742559; doi:10.3389/fimmu.2022.1047610)
Supplement: Supplementary file 3 [file DataSheet_3.pdf]

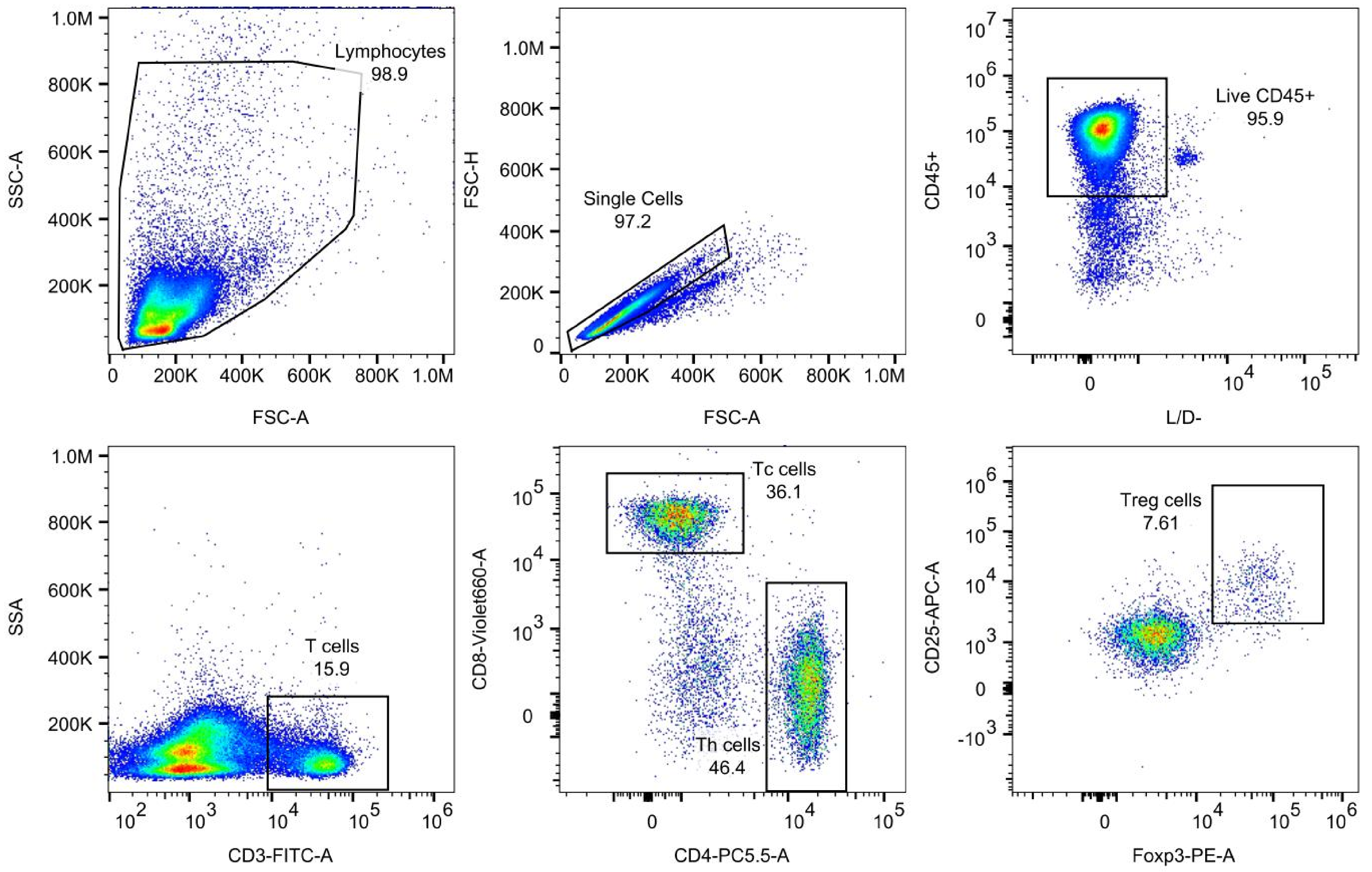
Lymphocytes
98.9
SSC-A
FSC-A
Single Cells
97.2
FSC-H
FSC-A
Live CD45+
95.9
CD45+
L/D-
T cells
15.9
SSA
CD3-FITC-A
Tc cells
36.1
Th cells
46.4
CD8-Violet660-A
CD4-PC5.5-A
Treg cells
7.61
CD25-APC-A
Foxp3-PE-A

IgG（6mg/kg）- 1

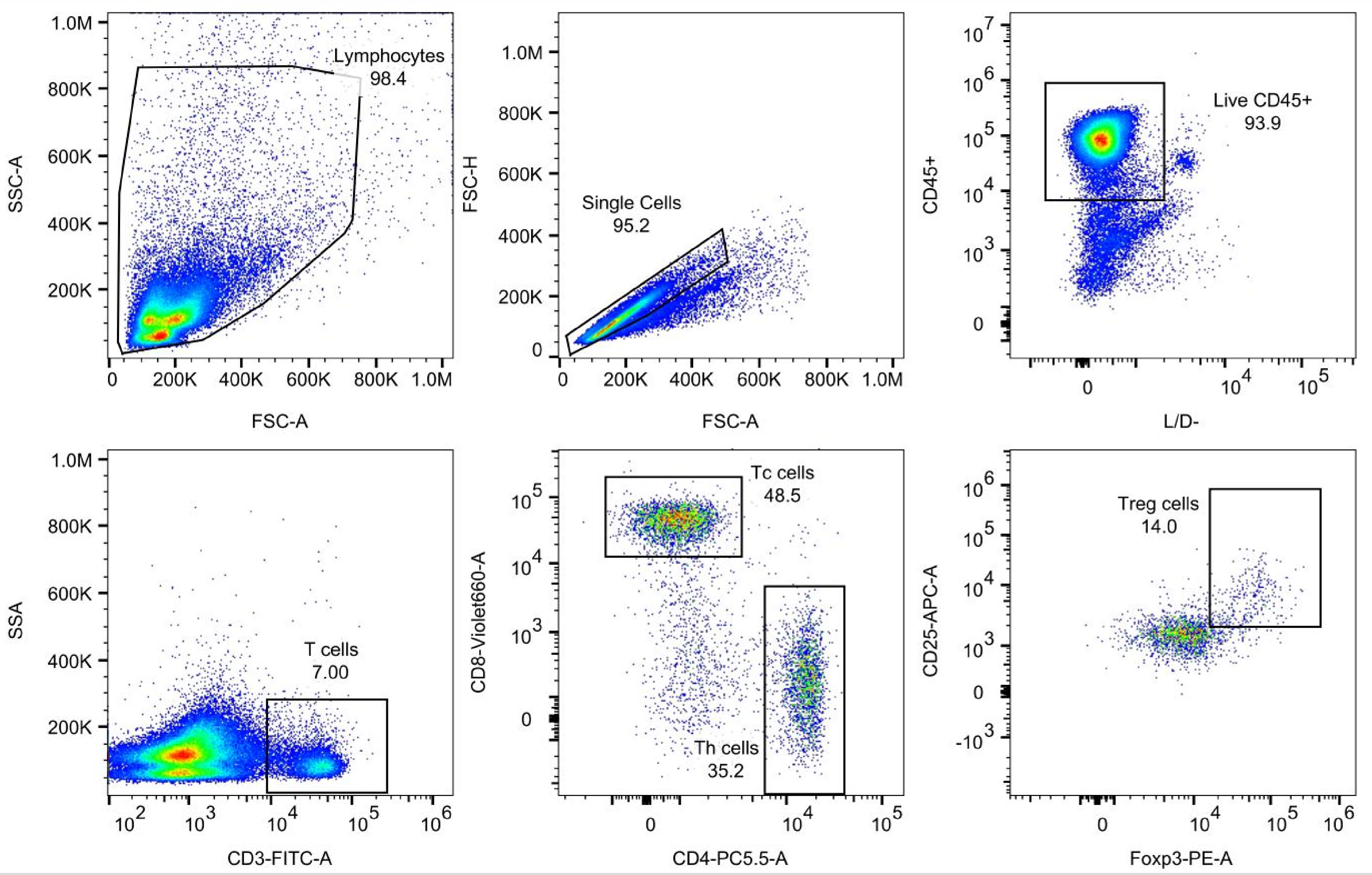
Lymphocytes
98.4
SSC-A
FSC-A
Single Cells
95.2
FSC-H
FSC-A
Live CD45+
93.9
CD45+
L/D-
T cells
7.00
SSA
CD3-FITC-A
Tc cells
48.5
Th cells
35.2
CD8-Violet660-A
CD4-PC5.5-A
Treg cells
14.0
CD25-APC-A
Foxp3-PE-A

IgG（6mg/kg）- 2

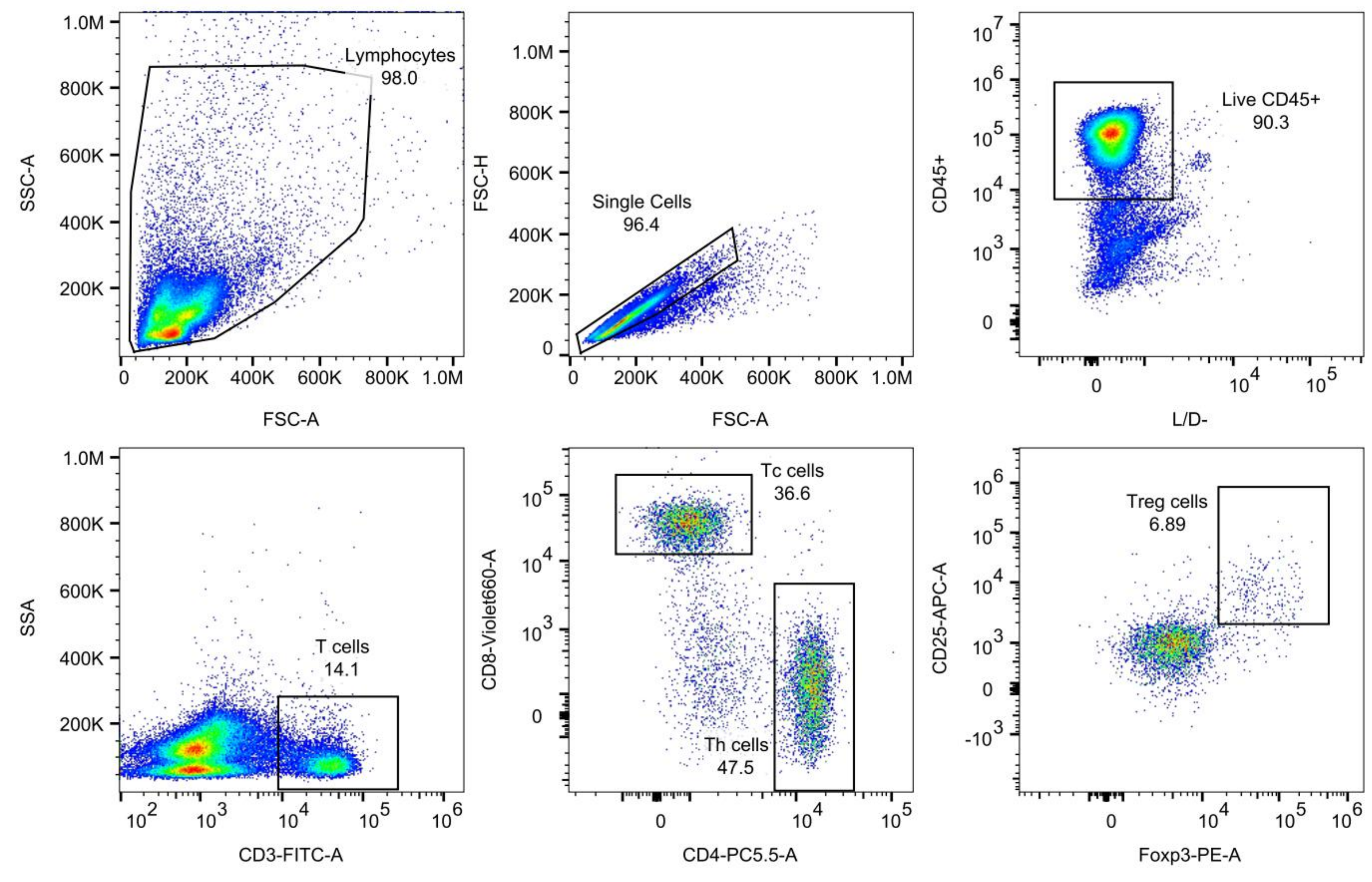

Lymphocytes
98.0
SSC-A
FSC-A
Single Cells
96.4
FSC-H
Live CD45+
90.3
CD45+
L/D-
T cells
14.1
SSA
CD3-FITC-A
Tc cells
36.6
Th cells
47.5
CD8-Violet660-A
CD4-PC5.5-A
Treg cells
6.89
CD25-APC-A
Foxp3-PE-A

IgG (6mg/kg) - 3

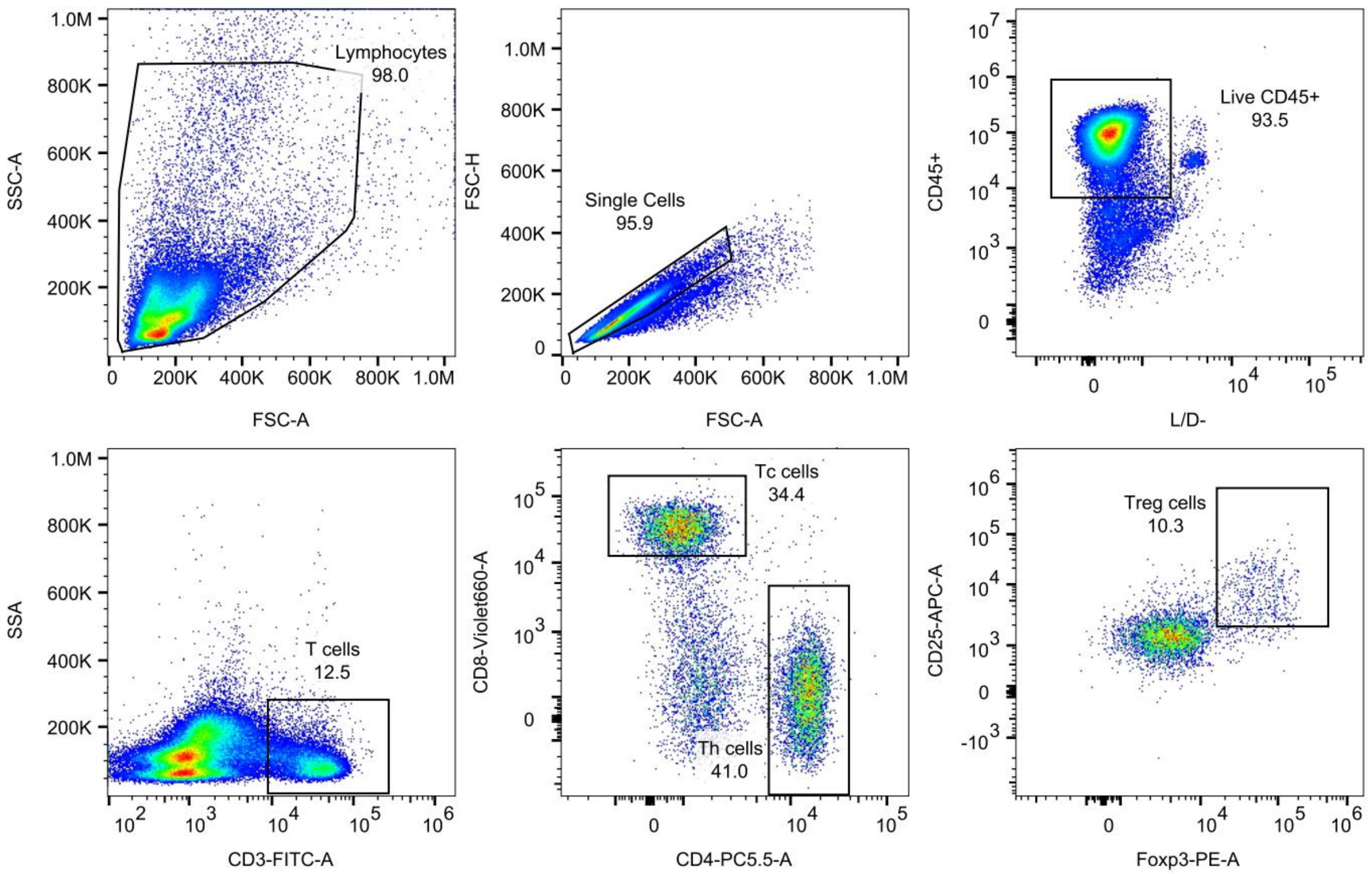
Lymphocytes
98.0
SSC-A
FSC-A
Single Cells
95.9
FSC-H
FSC-A
Live CD45+
93.5
CD45+
L/D-
T cells
12.5
SSA
CD3-FITC-A
Tc cells
34.4
Th cells
41.0
CD8-Violet660-A
CD4-PC5.5-A
Treg cells
10.3
CD25-APC-A
Foxp3-PE-A

IgG（6mg/kg）- 4

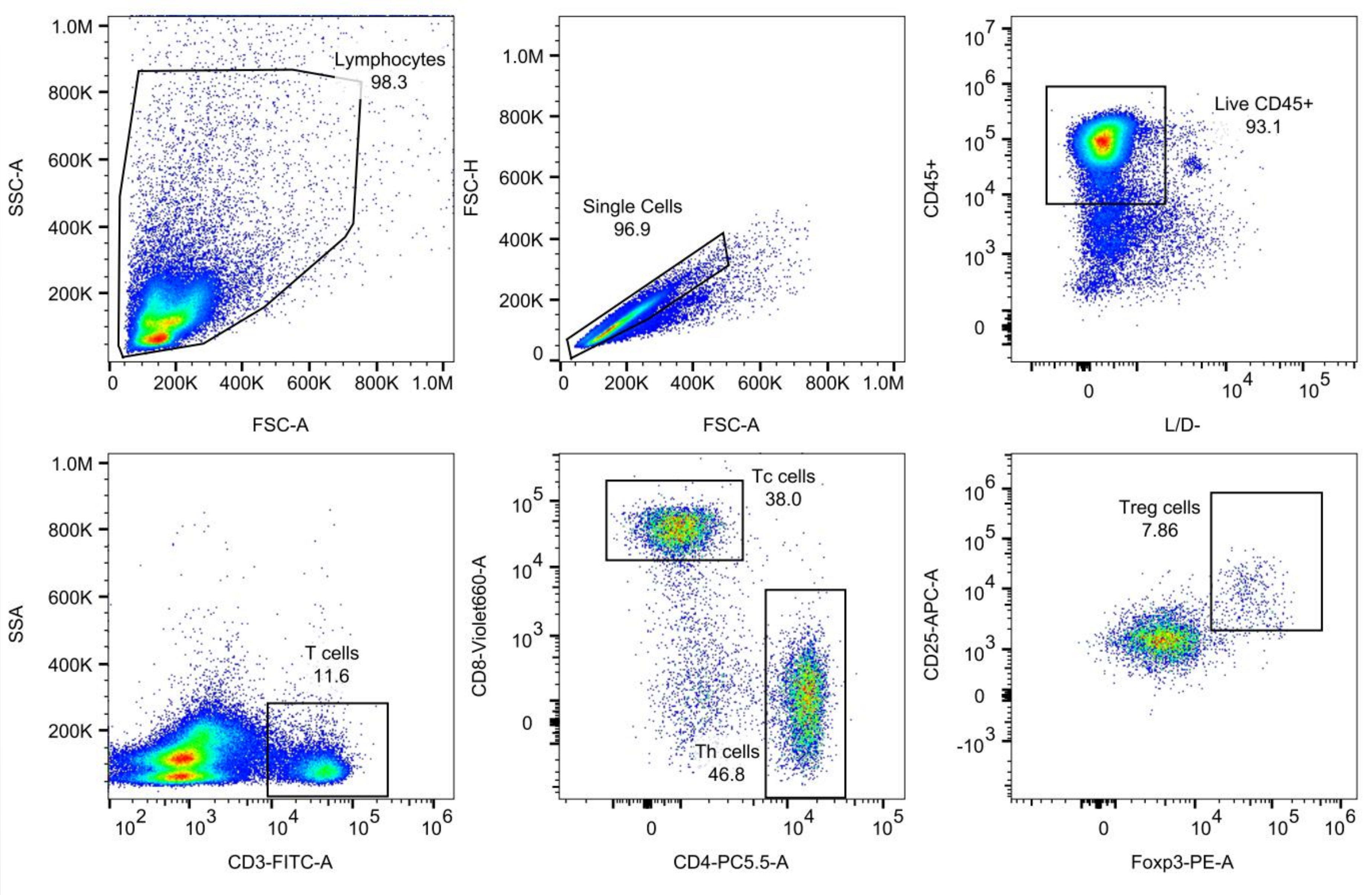
Lymphocytes
98.3
SSC-A
FSC-A
Single Cells
96.9
FSC-H
FSC-A
Live CD45+
93.1
CD45+
L/D-
T cells
11.6
SSA
CD3-FITC-A
Tc cells
38.0
Th cells
46.8
CD8-Violet660-A
CD4-PC5.5-A
Treg cells
7.86
CD25-APC-A
Foxp3-PE-A

IgG（6mg/kg）- 5

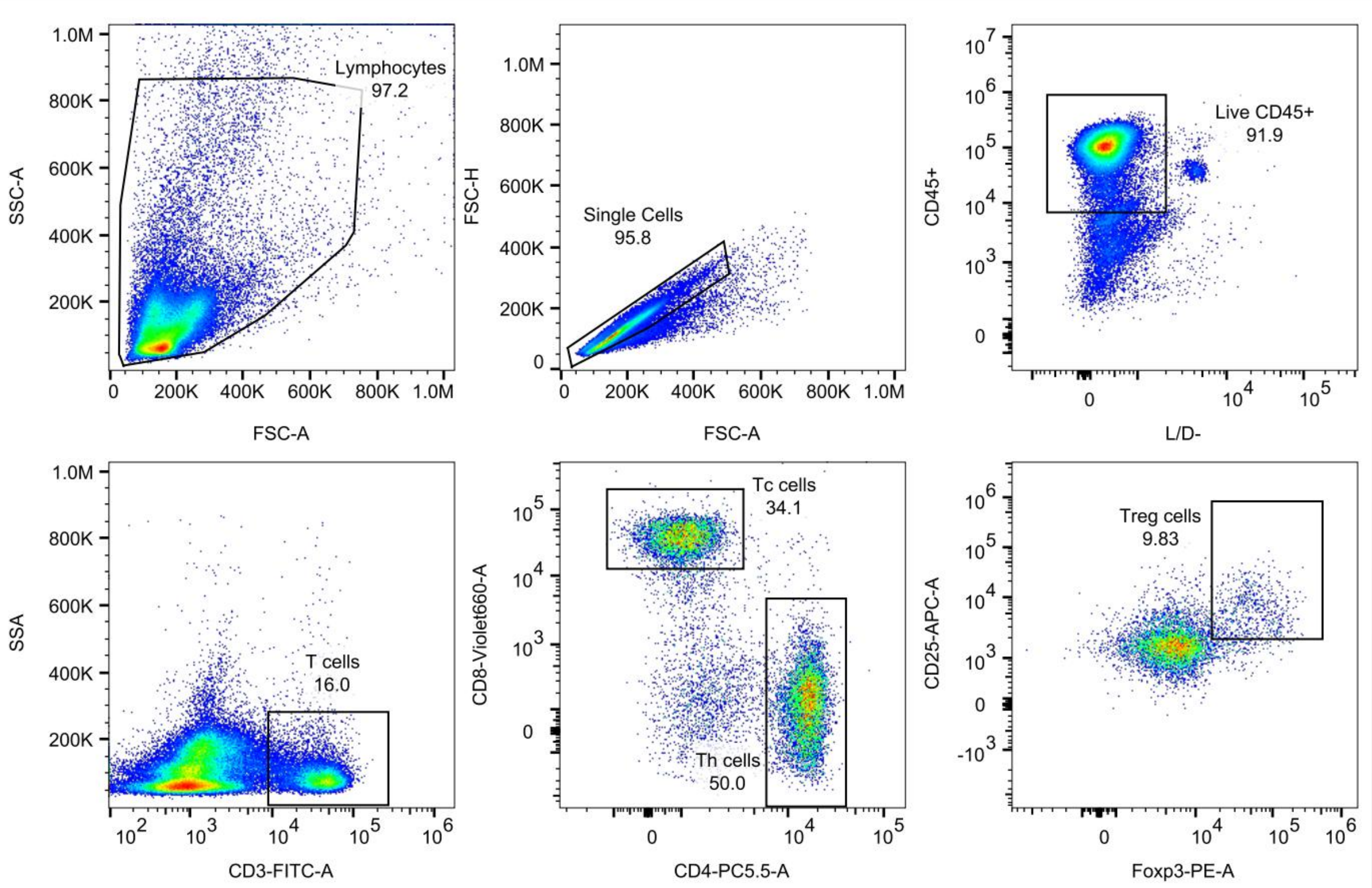
Lymphocytes
97.2
SSC-A
FSC-A
Single Cells
95.8
FSC-H
FSC-A
Live CD45+
91.9
CD45+
L/D-
T cells
16.0
SSA
CD3-FITC-A
Tc cells
34.1
Th cells
50.0
CD8-Violet660-A
CD4-PC5.5-A
Treg cells
9.83
CD25-APC-A
Foxp3-PE-A

IgG（6mg/kg）- 6

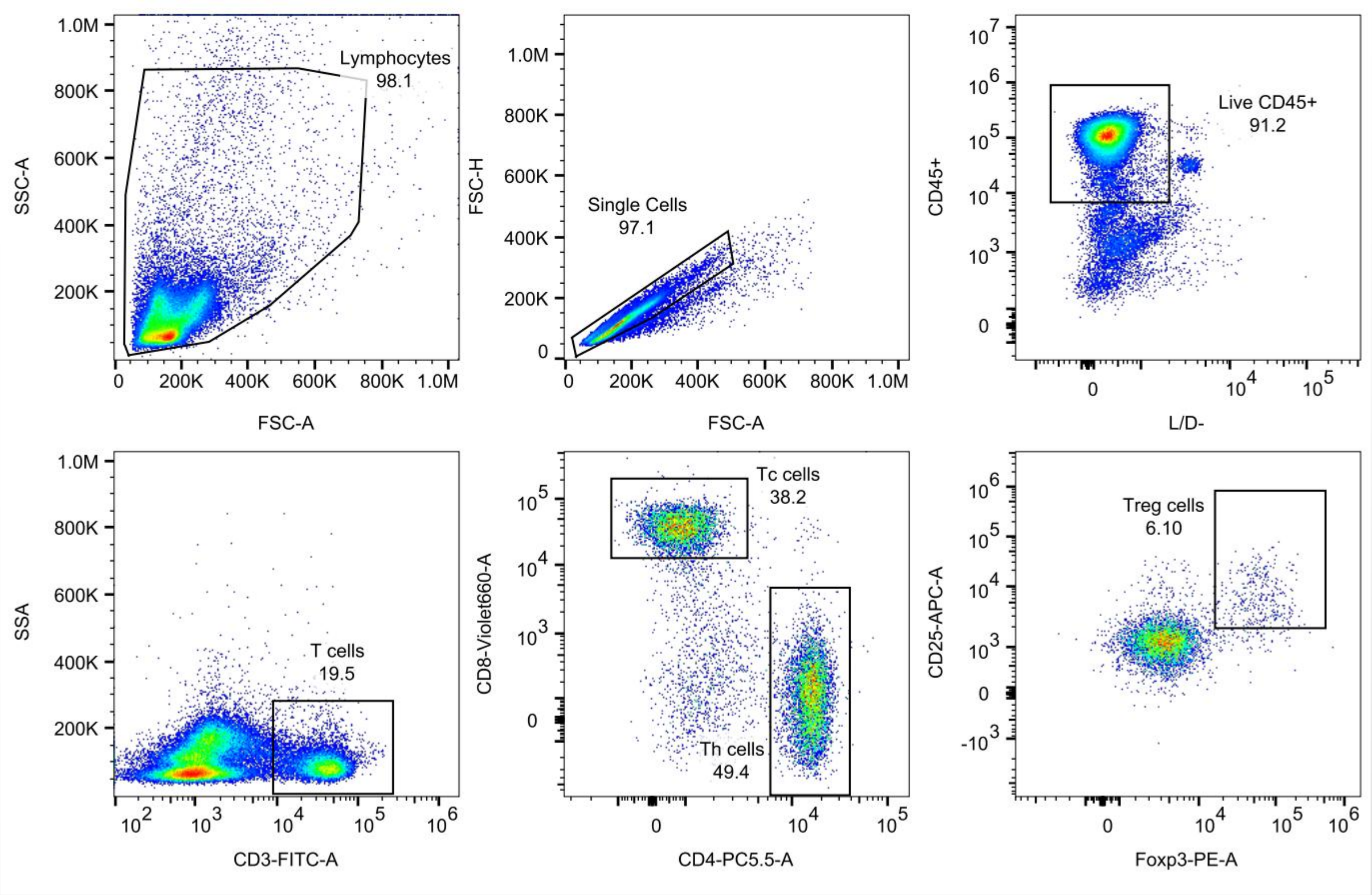

YG-003D3（2mg/kg） - 1

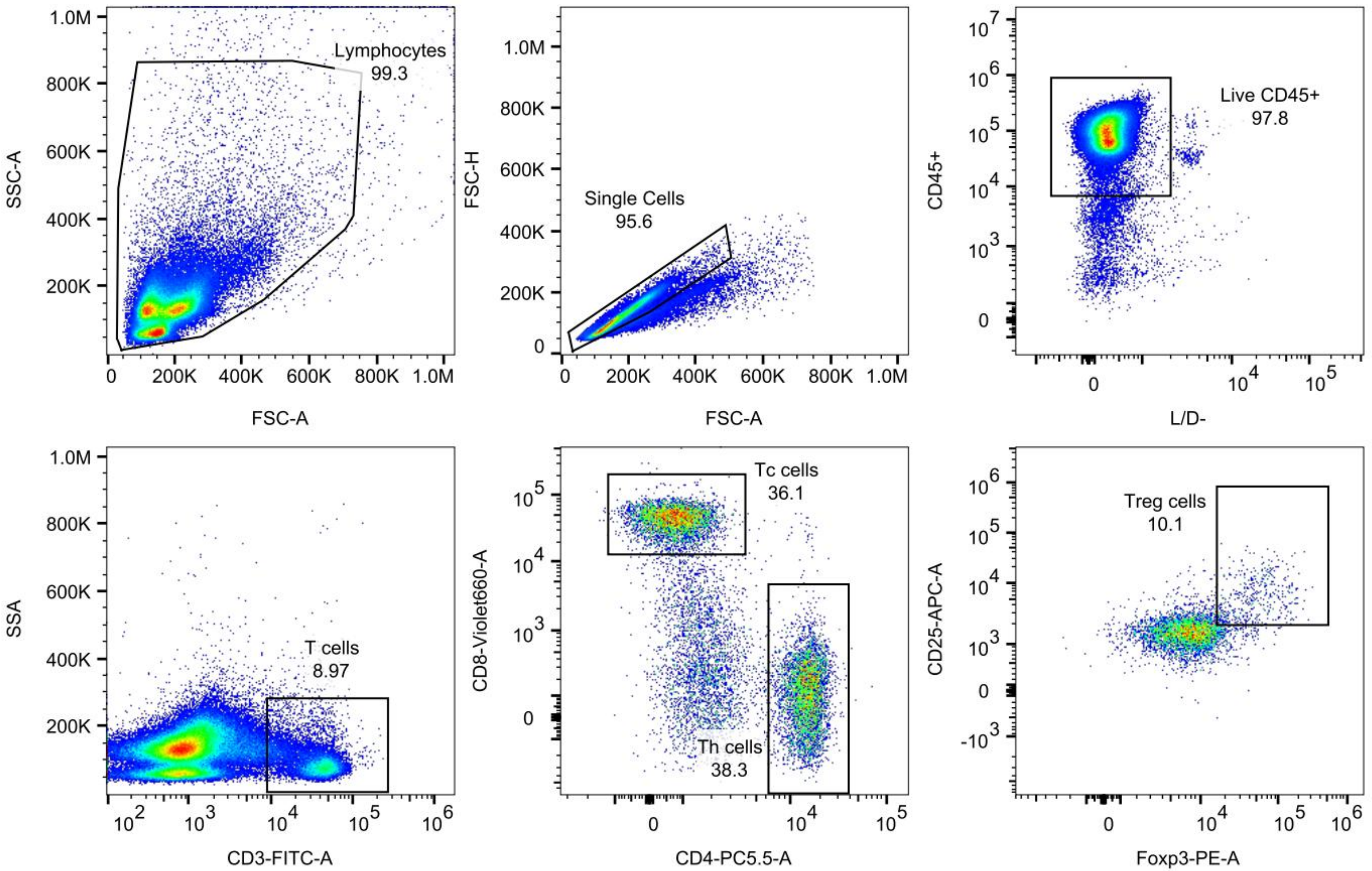

YG-003D3（2mg/kg） - 2

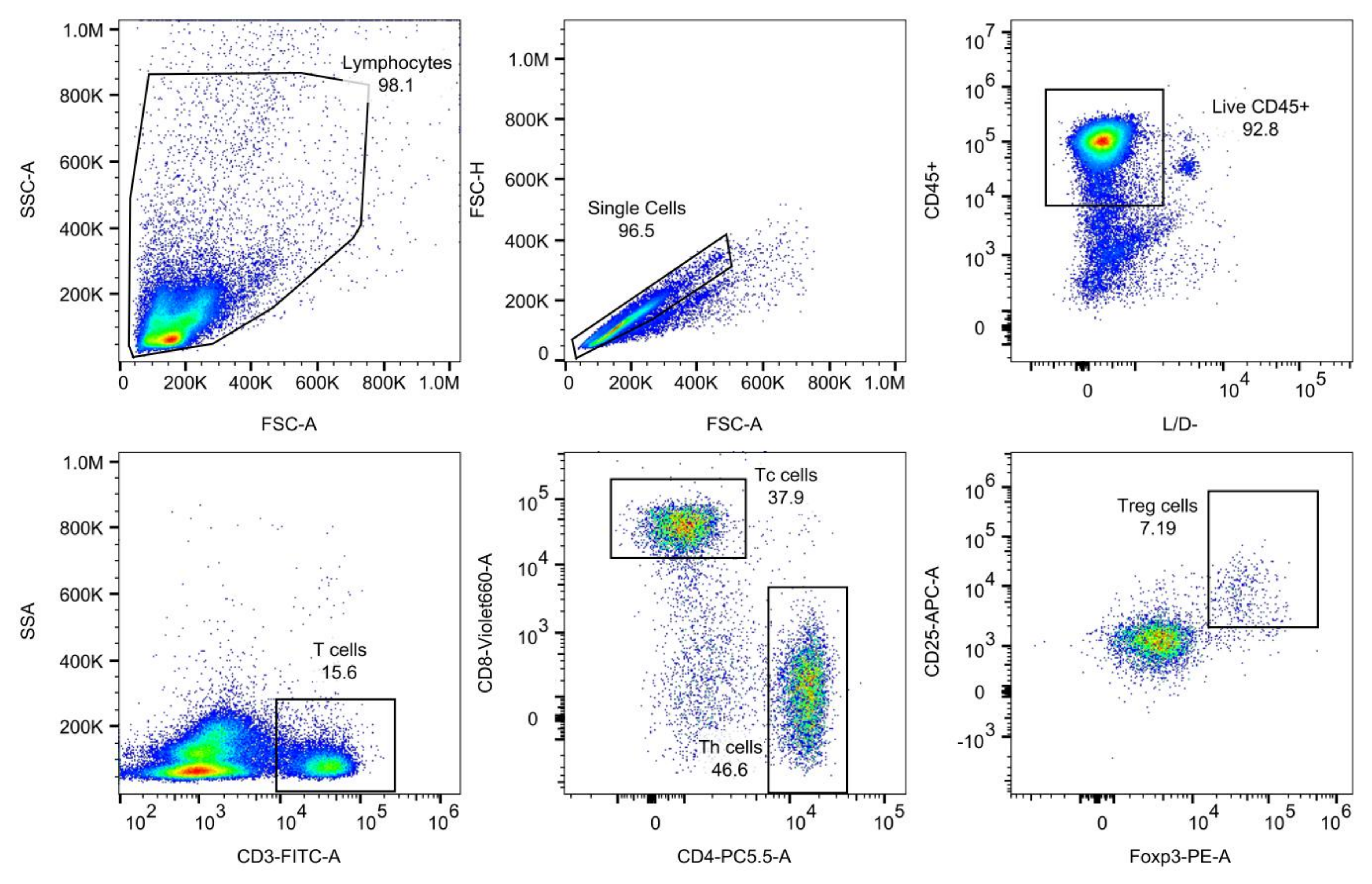

YG-003D3（2mg/kg）- 3

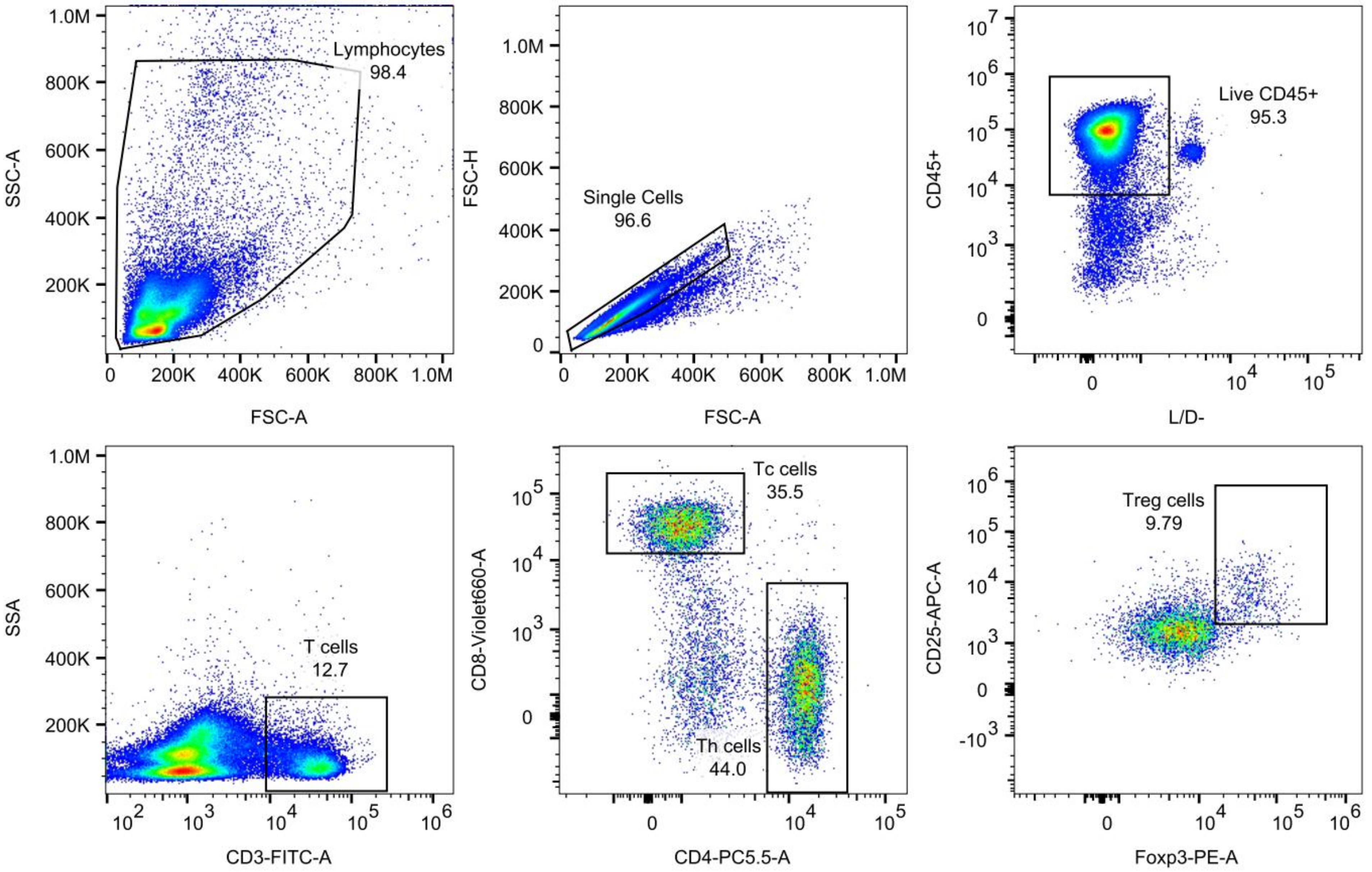

YG-003D3（2mg/kg）- 4

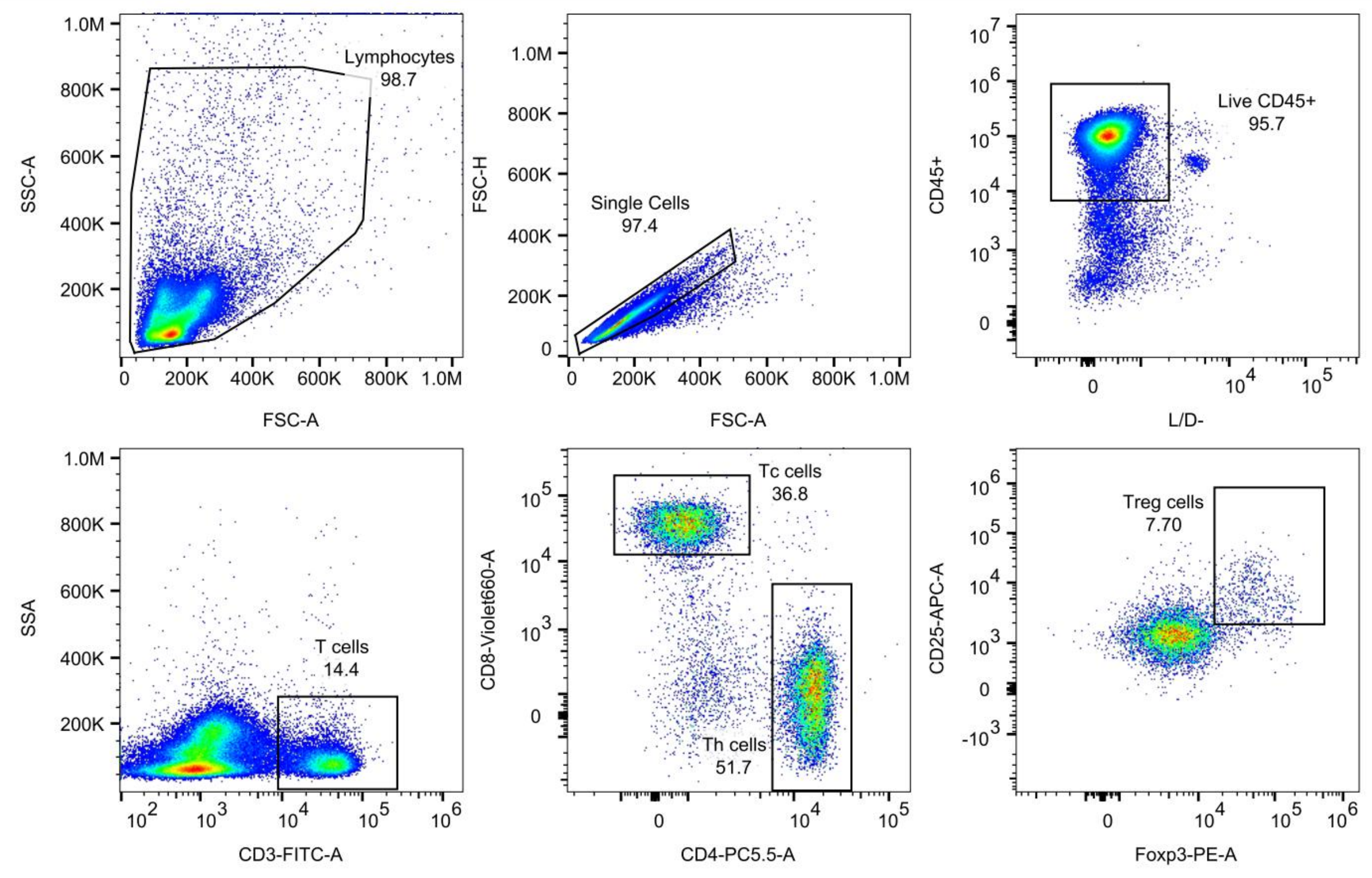

YG-003D3（2mg/kg）- 5

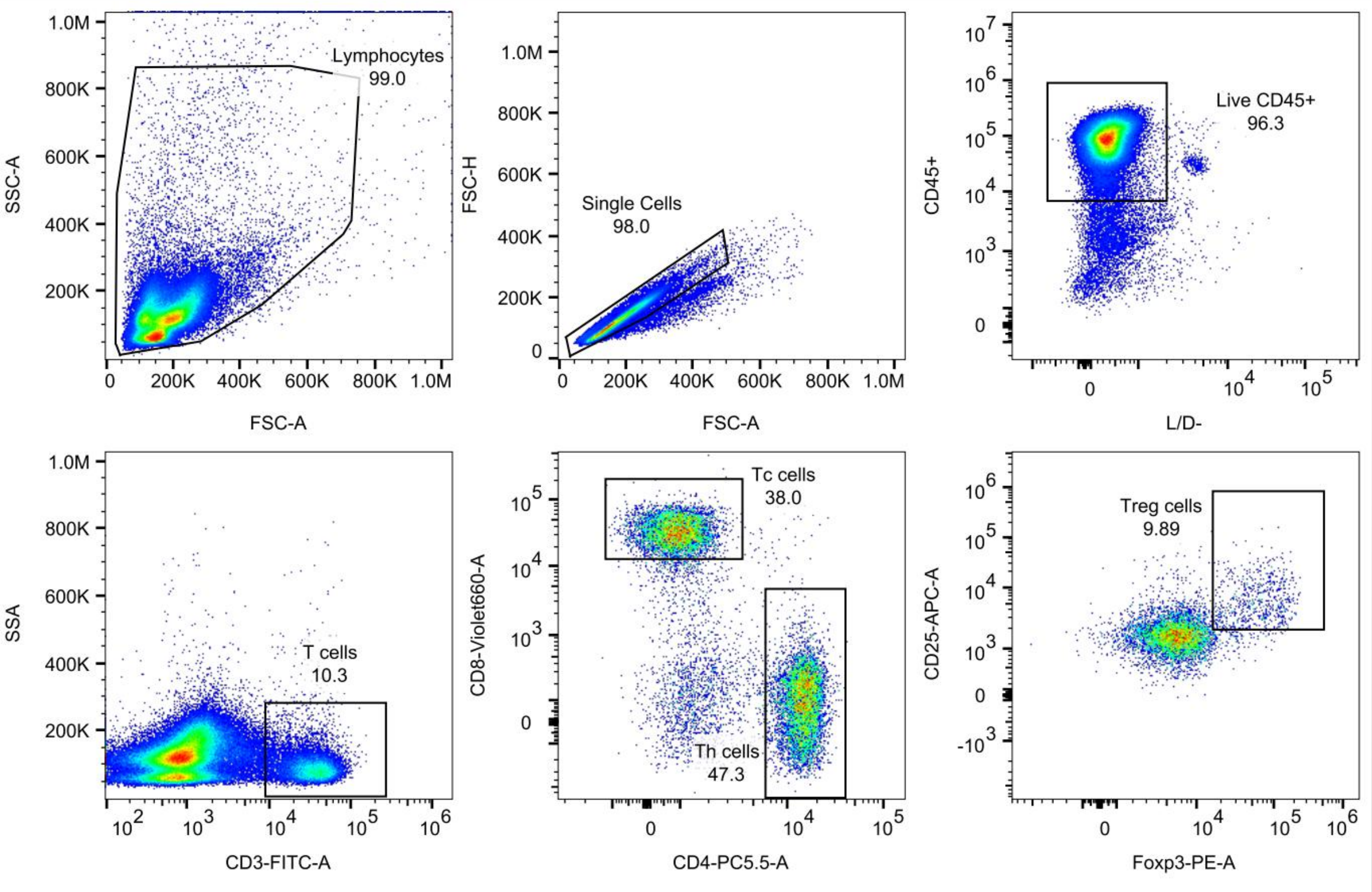

YG-003D3（2mg/kg）- 6

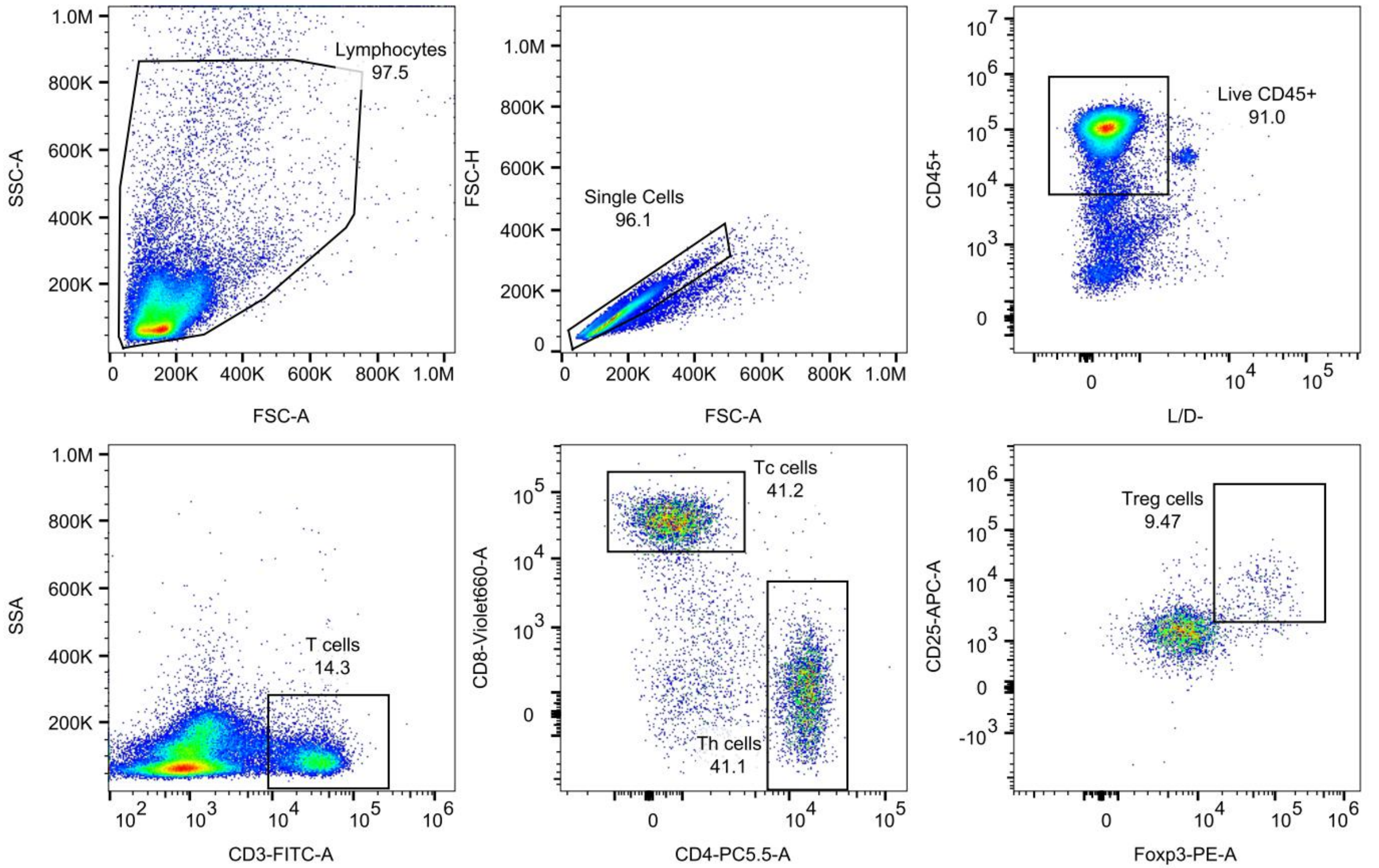

YG-003D3（6mg/kg）- 1

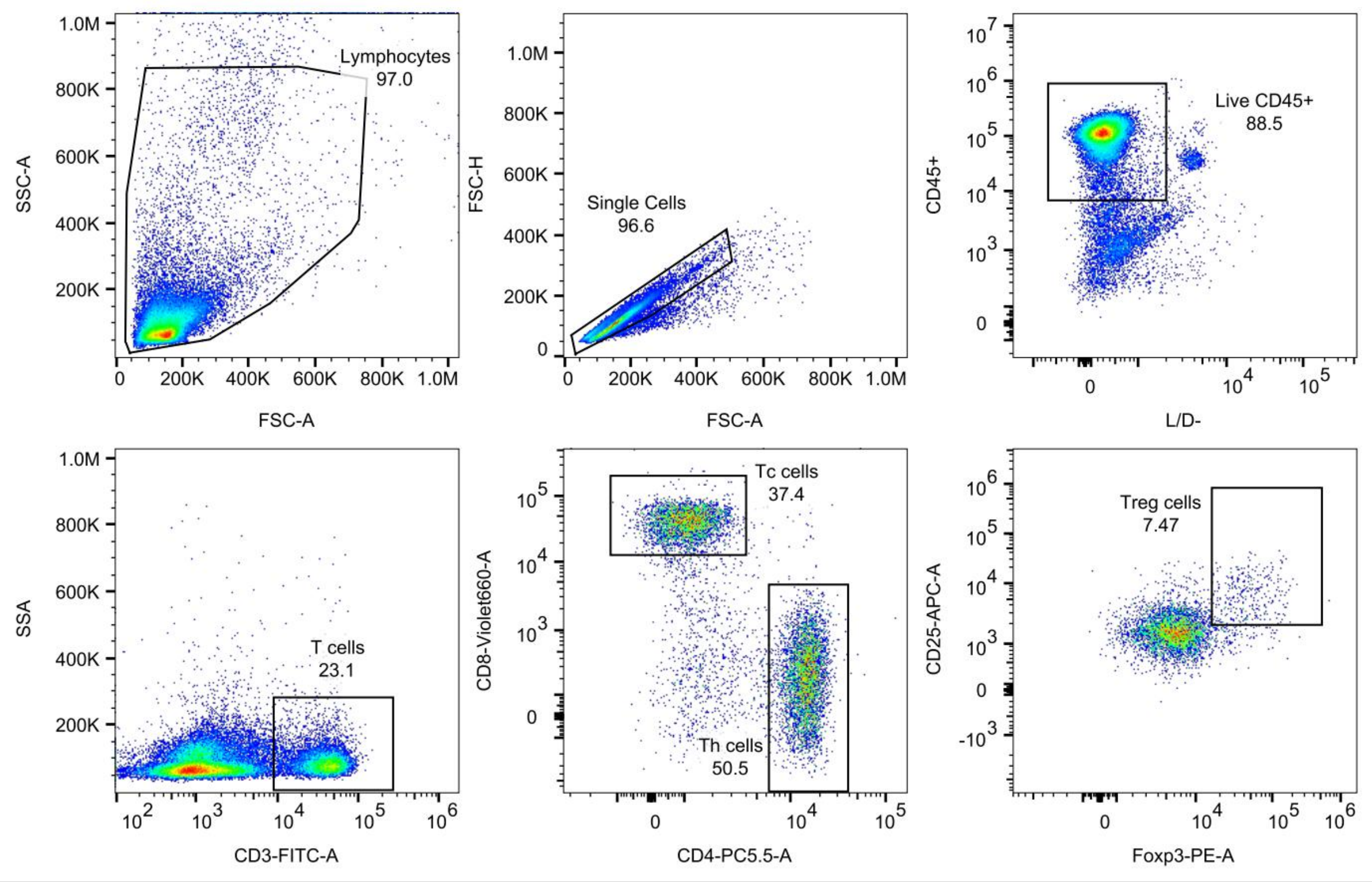

YG-003D3（6mg/kg）- 2

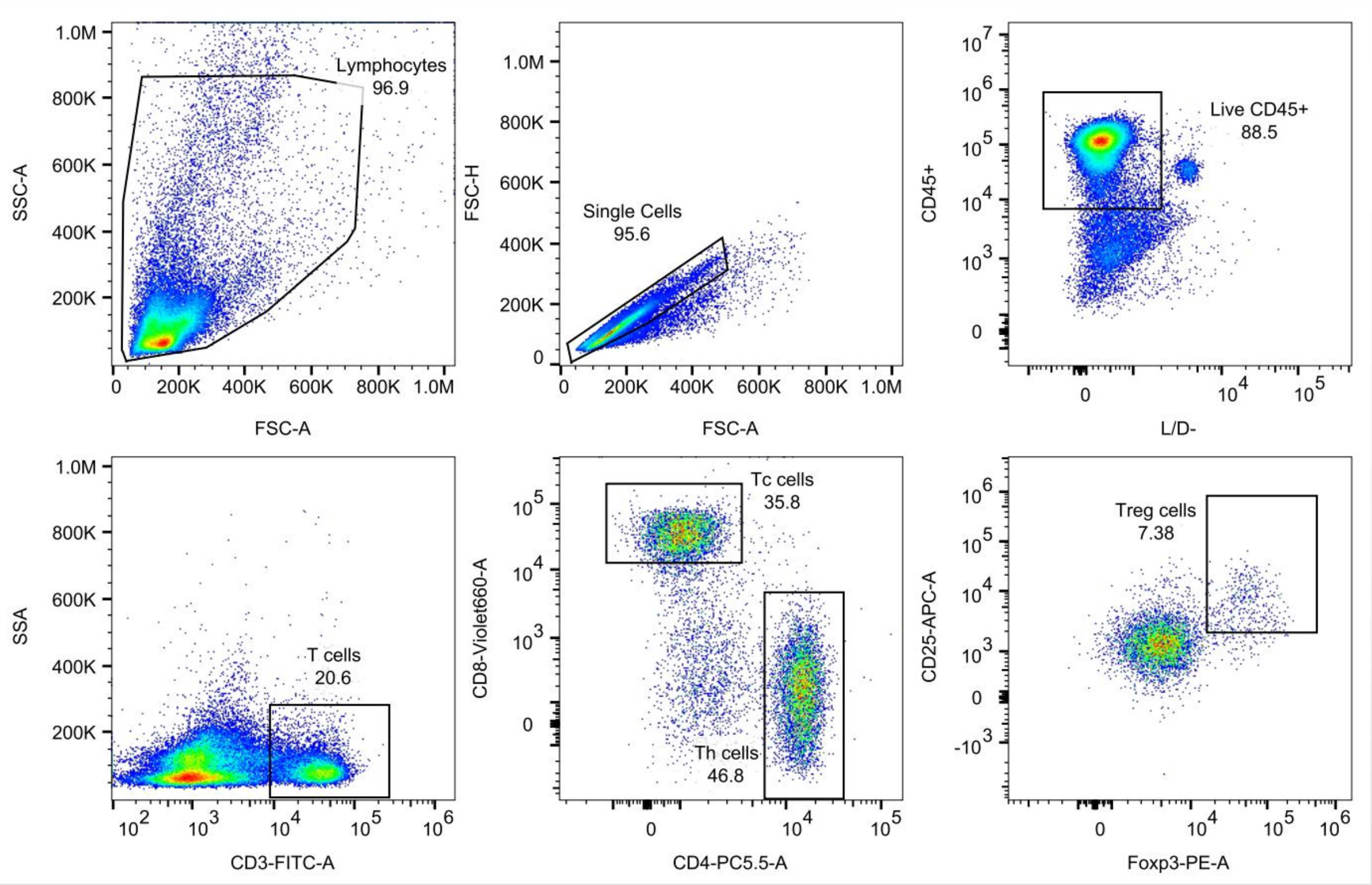

YG-003D3（6mg/kg）- 3

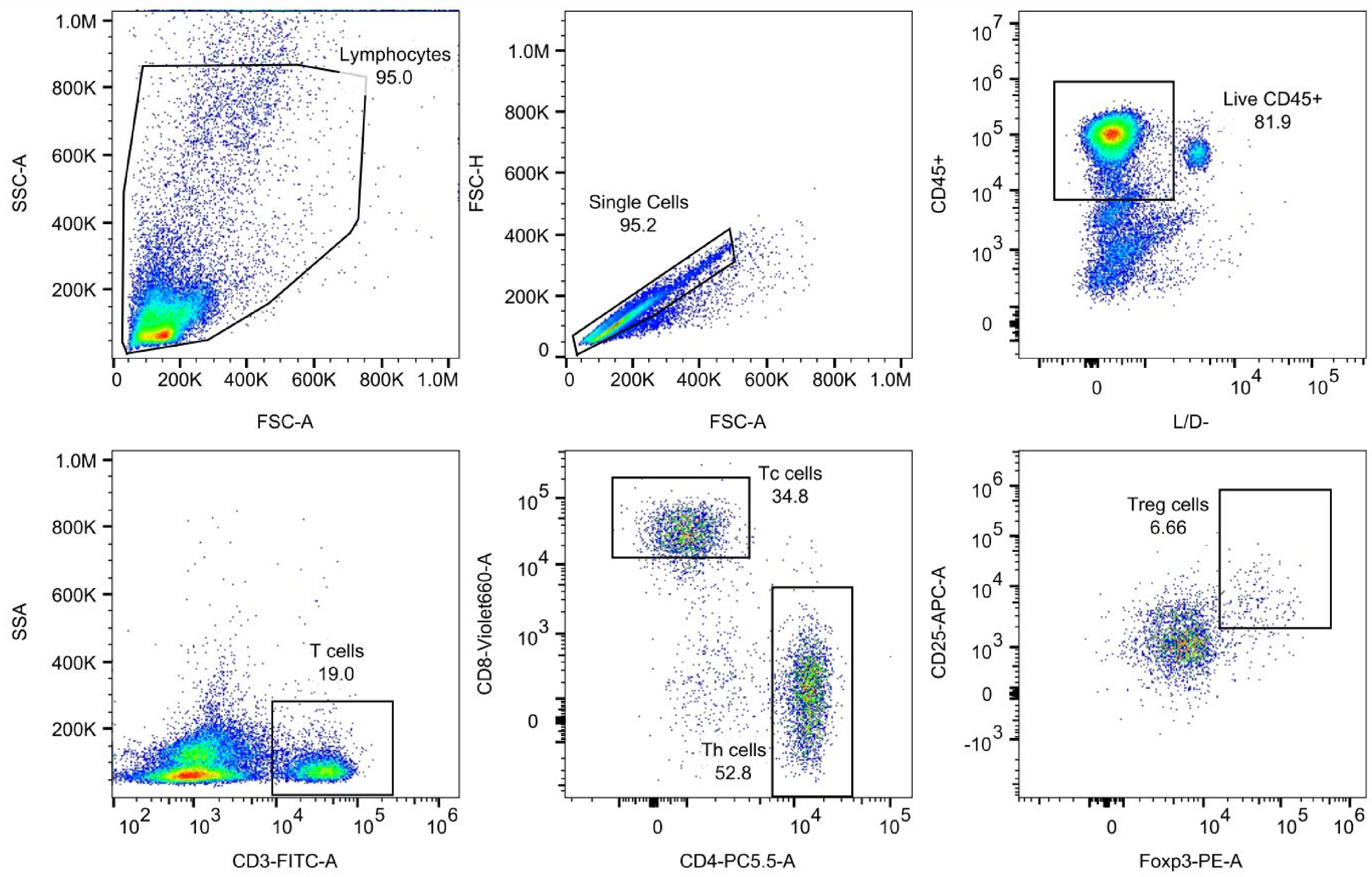

YG-003D3（6mg/kg）- 4

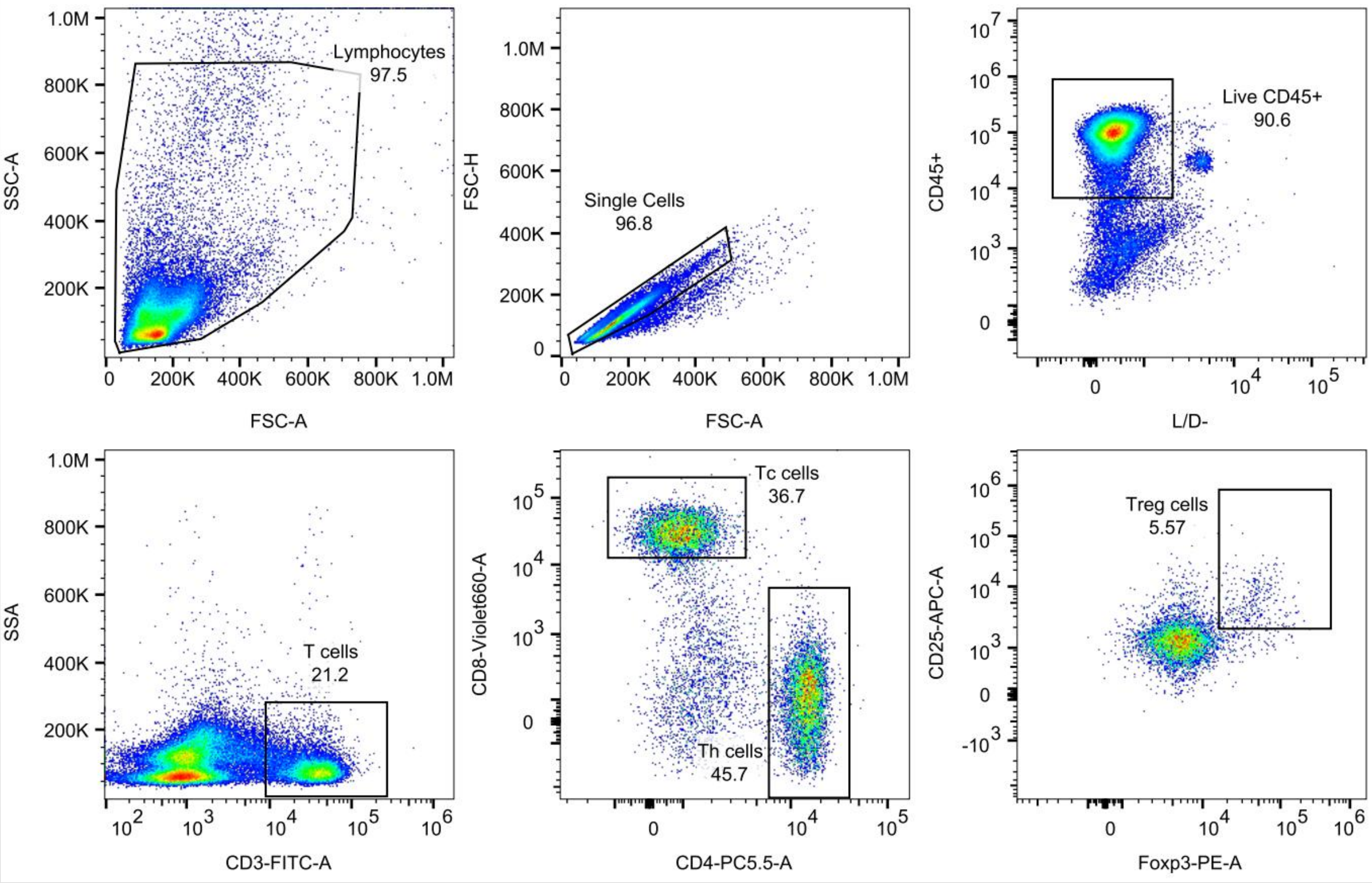

YG-003D3（6mg/kg）- 5

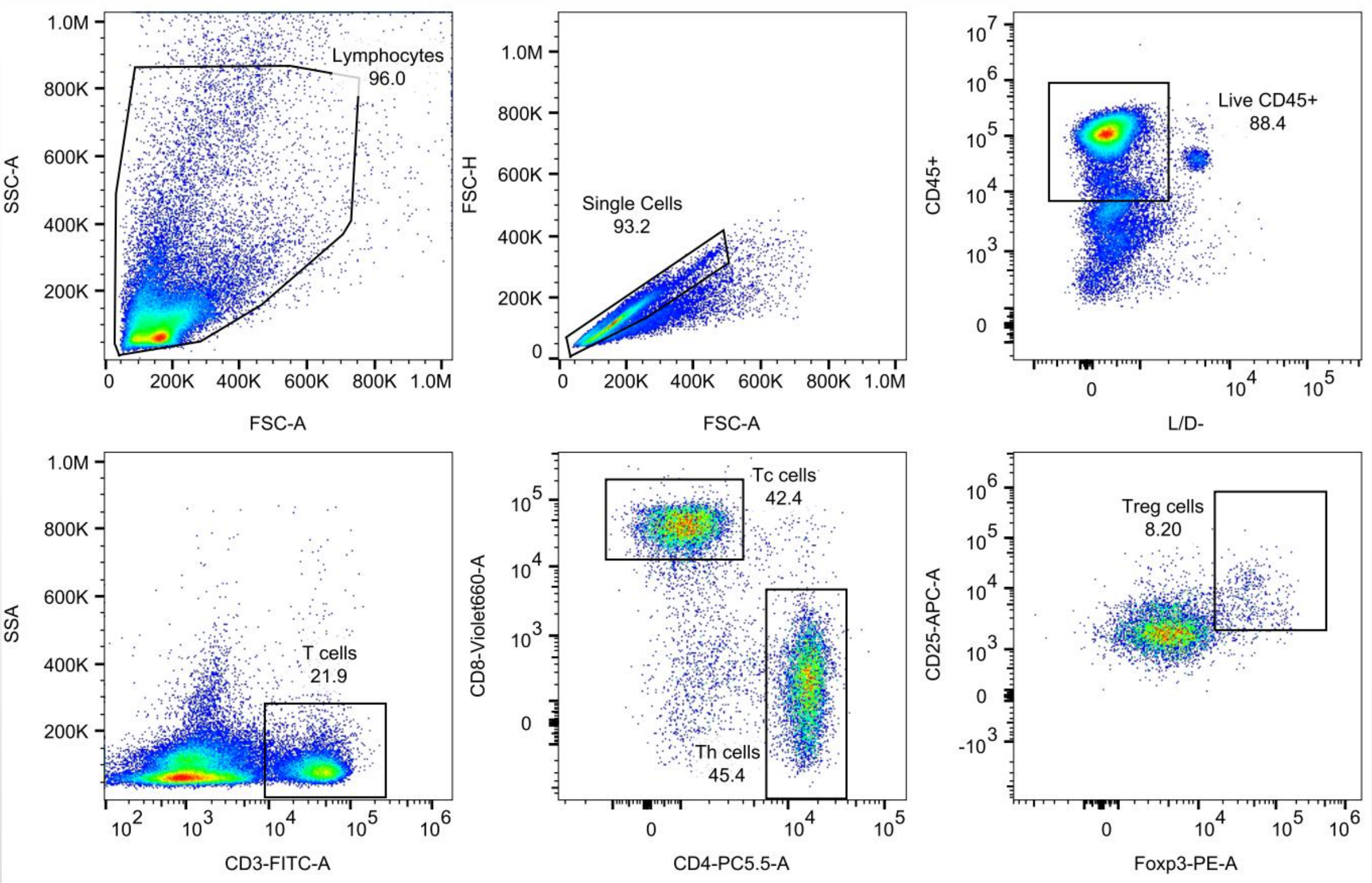

YG-003D3（6mg/kg） - 6
